# Femtosecond Plasmonic Laser Nanosurgery (fs-PLN) mediated by molecularly targeted gold nanospheres at ultra-low pulse fluences

**DOI:** 10.1038/s41598-020-68512-2

**Published:** 2020-07-24

**Authors:** Daniel Eversole, Kaushik Subramanian, Rick K. Harrison, Frederic Bourgeois, Anil Yuksel, Adela Ben-Yakar

**Affiliations:** 1 Biomedical Engineering, The University of Texas At Austin, Austin, TX 78712 USA; 2 Mechanical Engineering, The University of Texas At Austin, Austin, TX 78712 USA

**Keywords:** Cell delivery, Nanoparticles, Cancer imaging

## Abstract

Plasmonic Laser Nanosurgery (PLN) is a novel photomodification technique that exploits the near-field enhancement of femtosecond (fs) laser pulses in the vicinity of gold nanoparticles. While prior studies have shown the advantages of fs-PLN to modify cells, further reduction in the pulse fluence needed to initiate photomodification is crucial to facilitate deep–tissue treatments. This work presents an in-depth study of fs-PLN at ultra-low pulse fluences using 47 nm gold nanoparticles, conjugated to antibodies that target the epithelial growth factor receptor and excited off-resonance using 760 nm, 270 fs laser pulses at 80 MHz repetition rate. We find that fs-PLN can optoporate cellular membranes with pulse fluences as low as 1.3 mJ/cm^2^, up to two orders of magnitude lower than those used at lower repetition rates. Our results, corroborated by simulations of free-electron generation by particle photoemission and photoionization of the surrounding water, shed light on the off-resonance fs-PLN mechanism. We suggest that photo-chemical pathways likely drive cellular optoporation and cell damage at these off-resonance, low fluence, and high repetition rate fs-laser pulses, with clusters acting as local concentrators of ROS generation. We believe that the low fluence and highly localized ROS-mediated fs-PLN approach will enable targeted therapeutics and cancer treatment.

## Introduction

Precisely targeted femtosecond (fs) laser pulses have been used to disrupt cellular and sub-cellular structures with great accuracy for a variety of applications, including DNA and protein transfection across the cell membrane^1–4^, organelle manipulation^5,6^, axotomy^7^, and initiating controlled cell apoptosis^8,9^. However, the need for tight focusing to achieve nano-scale precision limits the number of structures that can be manipulated during therapy, restricting the technique to small-scale research applications.

In Plasmonic Laser Nanosurgery (PLN), the use of gold nanoparticles as local field enhancers holds promise as a scalable, low energy alternative to direct ultrashort (< 10 ps) laser irradiation for cellular manipulation^10,11^. The increased near-field strengths and/or large absorption cross-sections due to surface plasmons substantially reduce thresholds for photomodification. Plasmonic effects localize peak laser intensity in the particles’ vicinity, permitting more relaxed focusing conditions while maintaining nano-scale selectivity. We can also target molecularly-specific moieties with high selectivity^12^ since the surface of gold nanoparticles can be functionalized with bio-specific agents, such as antibodies^13^, ligands^14^, and DNA sequences^15^. These properties have the potential to reduce photo-treatment times and increase throughput, as large volumes of cells or tissue can be rapidly treated without needing for tight focusing on the desired target. More importantly, the substantial ablation threshold reduction would allow ablating deeper in a highly scattering tissue by reducing the probability of out-of-focus photodamage due to self-focusing.

These advantages have made PLN an effective strategy for cellular modification and transfection. The number and frequency of overlapping laser pulses govern how plasmonics influences the photomodification mechanism and intensity threshold. At low laser repetition rates (< 10 kHz), the extent of pulse-overlap usually remains low and pulse-to-pulse interactions are minimal. The large field enhancements around the nanoparticles induce ablation of nanoparticles^16^, and possibly bubble formation induced by the optical breakdown of the surrounding water, characterized by dense, reflective plasma (at electron densities greater than 1.7 × 10^21^ cm^−3^ at 800 nm)^17–27^. One or more of these processes likely drives the cell damage mechanism. These studies show an order of magnitude decrease in threshold pulse fluences compared to particle-free photodisruption^4–6,28–32^. Similar threshold reductions are seen for longer pulse widths (from 10′s of ps to up to a few ns), where thermal effects due to strong localized absorption play a dominant role^33–38^. As a side effect, however, the dense plasma formed in the femtosecond regime has been linked to cellular toxicity due to the resulting reactive oxygen species (ROS) generation that accompanies the bubble formation process^25,26^.

Higher laser repetition rates (> 2 MHz) are accompanied by a higher number of overlapping pulses and increased pulse-to-pulse interactions. Here, damage can occur via accumulative thermal and free-electron mediated photo-chemical damage regimes similar to conventional femtosecond laser surgery^28,39,40^. Fs-PLN studies in the 80-MHz-range have demonstrated photodamage at fluences an order of magnitude lower than that achieved for fs-PLN at kHz repetition rates^41,42^, while recent studies have even showed optoporation in an in vivo setting^43^. While the role of photothermal damage has been highlighted in the damage mechanism when using on-resonance nanoparticles^41^, the effect of free-electron mediated photo-chemical damage due to ROS has yet to be addressed at these high repetition rates.

In this paper, we explore the mechanism of nanoparticle-mediated photomodification at 80 MHz repetition rates using tightly focused, off-resonant femtosecond laser pulses at ultra-low fluences. We specifically investigate possible photo-chemical pathways to cellular optoporation and cell damage when using ~ 50 nm gold nanospheres. The particles act as biologically relevant, polarization independent local enhancers of laser intensity with minimal heating due to off-resonant excitation at 760 nm. We show that the PLN at this MHz repetition rate configuration occurs at an observable threshold fluence of approximately 1.3 mJ/cm^2^ per pulse, an order of magnitude lower than what has been previously observed with kHz repetition rates. In lieu of recent studies that have pointed to the role of clustering in the membrane permeabilization process^23,44,45^, we hypothesize that particle clustering plays a significant role in plasma membrane dysfunction and plasmonic laser optoporation. We show initial nanoparticle labeling concentration and epidermal grow factor receptor (EGFR) trafficking can drastically affect the outcomes of PLN by controlling particle clustering. Lastly, we present a comprehensive first-order model for particle photoemission and photoionization of the surrounding water to shed light on the photodisruption mechanism involved. Our results suggest a membrane dysfunction mechanism that is based on localized ROS formation with clusters acting as local concentrators of ROS generation and subsequent lipid peroxidation.

## Results and discussion

### Plasmonic laser nanosurgery threshold and optoporation efficiency

**Figure 1:**
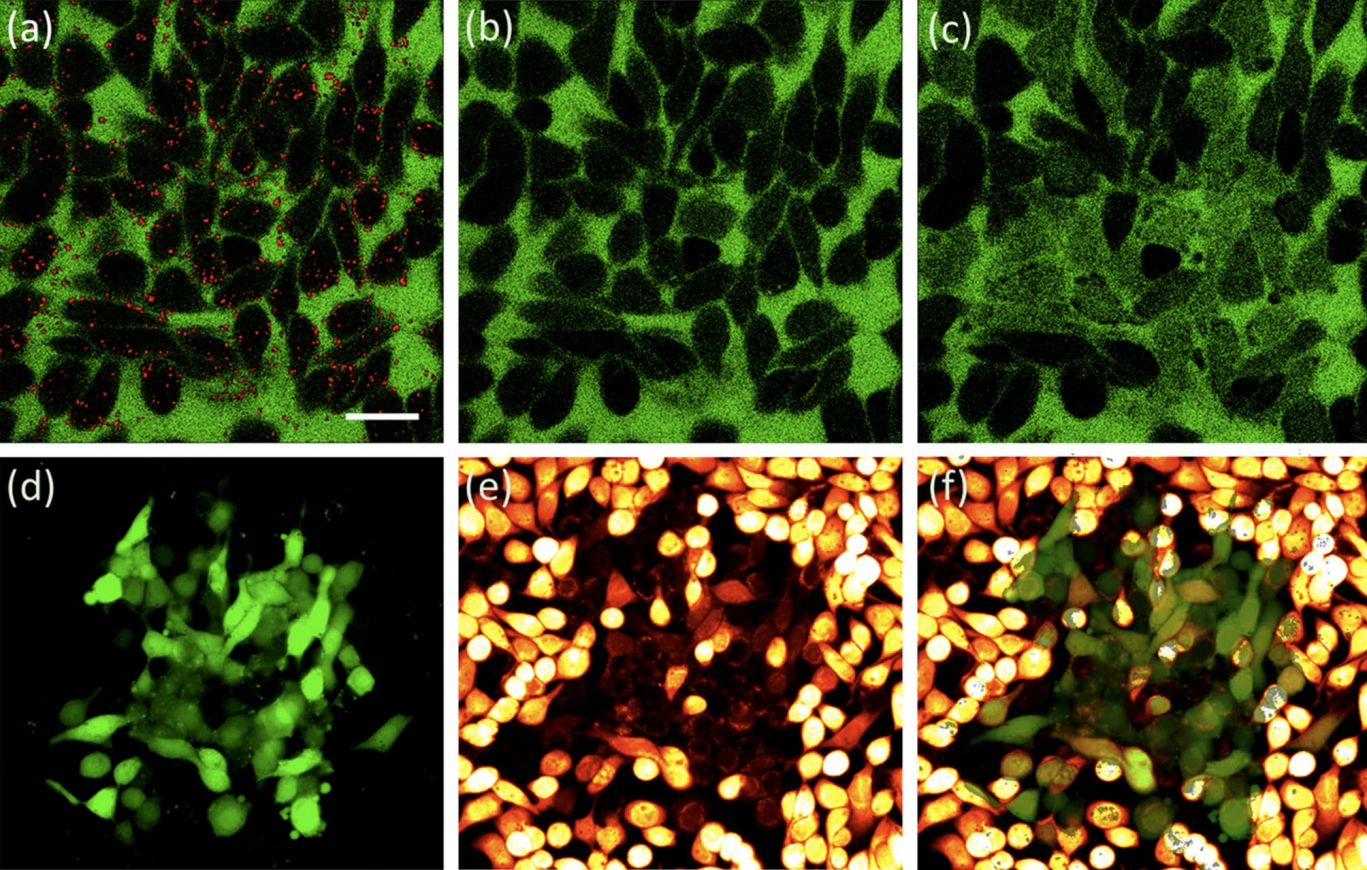
Plasmonic Laser Nanosurgery (PLN) of breast cancer cells as studied by the cellular intake of 10 kDa FITC-Dextran. (**a–c**) Multiphoton images showing FITC-Dextran in green (**a**) before, (**b**) immediately after, and (**c**) 5 min after PLN. A multiphoton luminescence image of labeled nanoparticles (red) is overlaid over two-photon fluorescence image of the FITC-Dextran (green) before PLN in (**a**). (**d–f**) Epifluorescence images showing FITC-Dextran retention and cellular viability to determine the PLN optoporation efficiency. The FOV was scanned 30 times with 2.6 mJ/cm^2^ average laser pulse fluence, delivering a total of 42,600 laser pulses to a single laser spot of 1.1 μm 1/e^2^ diameter. Images show (**d**) intracellular retention of 10 kDa FITC-Dextran, (**e**) cellular viability as probed with calcein red–orange AM, and (**f**) overlay of retention and viability images. The scale bar corresponds to 25 μm.

To find the fs-PLN threshold and efficiency, we studied the in vitro cell membrane optoporation of MDA-MB-468 triple-negative human epithelial breast cancer cells, labeled with 47 nm gold nanoparticles for different laser pulse fluences. We functionalized particle surfaces with antibodies that target EGFR, a membrane protein overexpressed by the breast cancer cells. For PLN experiments, we prepared monolayer constructs with nanoparticle-labeled cells. Before irradiating the cells with femtosecond laser pulses to perform PLN, we verified the distribution of gold nanoparticles along the cellular membrane using multiphoton luminescence microscopy^46^. PLN was then performed along the mid-plane of the monolayer cell constructs. Immediately following irradiation, two-photon microscopy was utilized to directly monitor the cellular intake of green fluorescent FITC-Dextran that was added into the extracellular solution prior to PLN. Cells were then washed and transferred to an upright microscope to monitor retention and viability 30 min post PLN. Short-term viability was assessed to determine the number of cells with intact membranes immediately post PLN, a sign of reversible membrane dysfunction. Long term viability post PLN itself was not the focus of our studies. Figure 1a–c show representative fluorescence images illustrating the entire PLN process as visualized with multiphoton microscopy. Figure 1d–f show example epifluorescence images of cells retaining intracellular FITC-Dextran after exposure to an average pulse fluence of 2.6 mJ/cm^2^ after 30 full scans of the field of view (FOV). Viable cells are indicated by calcein red–orange fluorescence. A sharp boundary delineates green (FITC-Dextran) optoporated cells from non-optoporated cells. Dead cells are visualized as ghosts, only exhibiting weak calcein red–orange fluorescence along the dysfunctional bilipid membrane.

To determine the fs-PLN threshold and efficiency, we performed 30 full scans at average laser powers varying between 1 and 7 mW, corresponding to laser pulse fluences of 1.3 and 9.1 mJ/cm^2^ and peak irradiances of 4.8 and 33.6 GW/cm^2^. Henceforth, all references to pulse fluence will refer to average fluence per pulse, unless otherwise stated. One full scan of the 150 × 150 μm^2^ FOV took about 330 ms. Considering the measured 1/e^2^ beam diameter of 1.1 ± 0.2 μm, the dwell time over one laser spot during one line-scan was approximately 4.7 µs, resulting in 378 consecutive pulses overlapping per spot, and a total of ~ 1,420 pulses after one full FOV scan and ~ 42,600 pulses after 30 full FOV scans.

**Figure 2:**
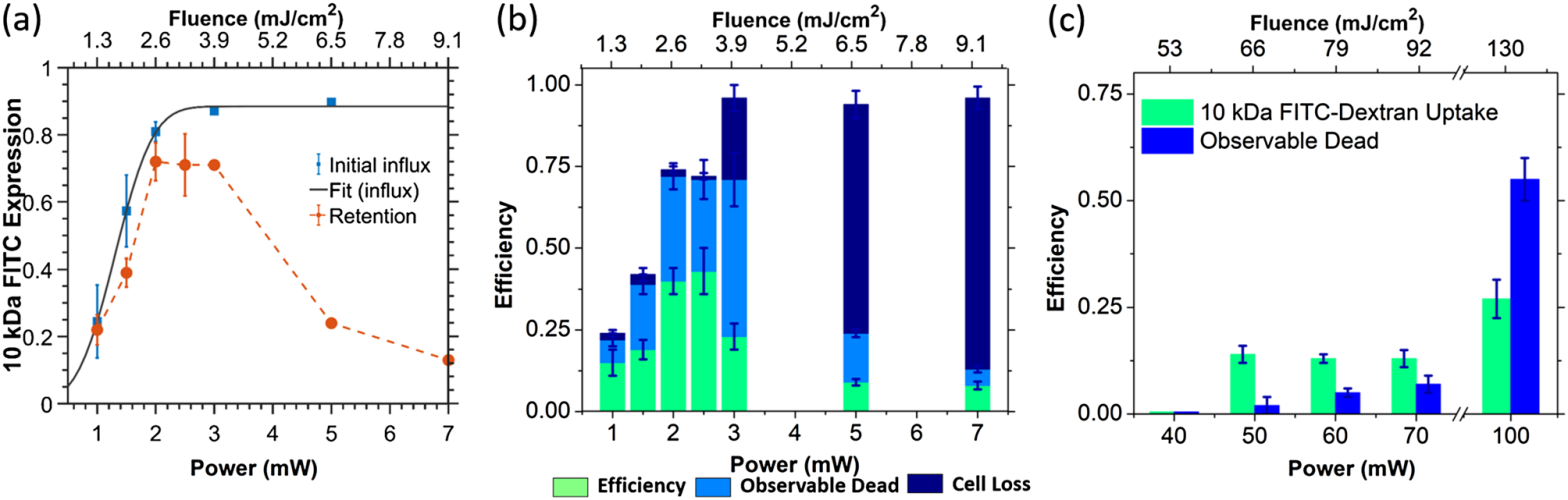
Membrane optoporation efficiency for uptake of 10 kDa FITC-Dextran in labeled and unlabeled cells. (**a**) Plots show the percentage of cells incurring (5 min after PLN) and retaining FITC-Dextran influx (30 min after PLN and washing of the extracellular FITC-Dextran) with respect to average laser power and pulse fluence as detected with two-photon fluorescence imaging. The solid line shows the fit to Eq. 1 for the initial influx data, depicting the underlying normal distribution. (**b**) The optoporation efficiency as determined using epifluorescence images for FITC-Dextran retention and cell viability (30 min after PLN). (**c**) The percentage of unlabeled cells retaining FITC-Dextran influx and their viability after PLN with varying average laser pulse fluences. All error bars represent the standard error of the mean for n ≥ 7 independent experiments for (**a**) and (**b**), and n = 4 for (**c**). Cells were labeled at a concentration of 20,000 particles per cell and both labeled and unlabeled cells were irradiated for 30 scans.

Figure 2a shows the percentage of cells incurring and retaining 10 kDa FITC-Dextran influx with respect to laser fluence as measured 5 and 30 min after laser irradiation using two-photon and epifluorescence imaging, respectively. The lowest pulse fluence at which we could detect FITC-Dextran influx was 1.3 mJ/cm^2^. The number of cells exhibiting FITC-Dextran influx rapidly increased with laser fluence, plateauing near 90% for laser pulse fluences of 9.1 mJ/cm^2^ and higher. Maximum FITC-Dextran retention (73 ± 4%) was observed at 2.6 mJ/cm^2^ as measured using epifluorescence imaging 30 min after PLN and after the extracellular FITC-Dextran was washed away with DPBS. Beyond 3.9 mJ/cm^2^, the retention rates began to drop rapidly indicating irreversible membrane damage.

The efficiency of cellular optoporation was defined as the percentage of cells that both retained the fluorescent FITC-Dextran and remained viable. The optoporation efficiency reached 43 ± 7% at 2.6 mJ/cm^2^ but was lower than the FITC-Dextran retention due to cellular death (Fig. 2b). Rapidly increasing cellular loss and death at higher pulse fluences further reduced the overall optoporation efficiency. Above 6.5 mJ/cm^2^, membrane damage over the entire optoporated cell population became irreversible, with 90 ± 5% cell death and negligible optoporation efficiencies. It should be noted that across the fluences studied, PLN never produced 100% cellular death, with the highest pulse fluence of 9.1 mJ/cm^2^ approximately resulting in 5–10% viability. At pulse fluences 3.9 mJ/cm^2^ and lower, only a small percentage of cells were removed after the washing steps.

In comparison, unlabeled cells subjected to the same PLN process showed a 50-fold increase in threshold pulse fluence needed to detect FITC-Dextran influx (Fig. 2c). Below this threshold (65 mJ/cm^2^), unlabeled cells remained unaffected, i.e. viable with no FITC-Dextran influx. For fluences above the threshold, the number of unlabeled cells exhibiting FITC-Dextran influx remained nearly constant, with 55% of unlabeled cells rendered nonviable at the maximum tested pulse fluence of 130 mJ/cm^2^ (100 mW).

The optoporation events in the labeled cells increase gradually with increasing fluence, rather than the step wise change observed at a “threshold fluence” in unlabeled cell. This gradual increase in labeled cells cannot be explained solely by the slight ellipticity of the nanoparticles, as the measured aspect ratio of 1.24 ± 0.13 (Supplementary Fig. 1, Supplementary Note 1) was not substantial enough to show a discernable second peak in the extinction spectrum. A bigger contribution to the gradual increase, most probably, stems from the possible clustering of nanoparticles, a process known to occur in EGFR-conjugated nanoparticles^13,47^. The varying particle geometries and their cluster sizes cause different cells to experience different levels of disruption for a given pulse fluence. If nanoparticles are assumed to randomly cluster on the cell surface, a normal distribution of particle numbers and geometries, and thus local disruption rates exist on the cellular surface over the whole cell population. We can then calculate the fluence at the point where 50% of the cells experience FITC-Dextran influx, $F_{\mu }$, by fitting an error function to the initial influx data points given in Fig. 2a, derived from the underlying normal distribution,

1 $$\varphi_{FITC} = \frac{{\varphi_{FITC}^{\infty } }}{2}.\left[ {1 + {\text{erf}}\left( {\frac{{F_{av} - F_{\mu } }}{\sqrt 2 \sigma }} \right)} \right]$$

Here, $\varphi_{FITC}$ represents the percentage of cells incurring FITC, $\varphi_{FITC}^{\infty }$ is the maximum percentage of achievable FITC-Dextran uptake, and *F* the incident pulse fluence. The standard deviation, *σ*, represents the range of fluences centered at $F_{\mu }$ over which 68% of the cells are optoporated, and can be interpreted as the measure of the variability introduced by nanoparticle clustering. The fit yielded a mean fluence of $F_{\mu }$ = 1.72 ± 0.06 mJ/cm^2^ for the uptake of 10 kDa FITC Dextran and *σ* = 0.69 ± 0.10 mJ/cm^2^. A fit of Eq. (1) to initial influx data (Fig. 2a) estimated the PLN optoporation threshold to be *F*_*th*_≈0.36 ± 0.12 mJ/cm^2^ as the point where 4.6% (2*σ*) of the cells are optoporated.

Shifts in propagation of cell population, aging nanoparticle batches, and antibody batch variations have the potential to limit experimental control over time. To ensure PLN repeatability over the duration of data collection, a two-tailed *t*-test having equal variance was performed on recorded observable cellular death and optoporation efficiencies. Resultant p-values were greater than 0.5, indicating negligible differences in observable death and optoporation efficiencies in different experiments recorded over time.

### Characterization of particle clustering effects

Since EGFR are reported to be heterogeneously distributed on the cell surface^48,49^, individual nanoparticles are expected to cluster as they bind at the concentrated EGFR sites. To understand how particle clustering might affect cellular membrane dysfunction, we varied the spatial localization of the nanoparticles and measured its effects on optoporation efficiency. Spatial localization of the nanoparticles on the cell surface was altered by changing the labeling concentration and labeling temperature. Permeabilization with linearly and circularly polarized laser pulses is also compared to glean a better understanding of the overall geometry of the clusters formed and how they influence membrane dysfunction efficiency.

#### Effects of labeling concentration

**Figure 3:**
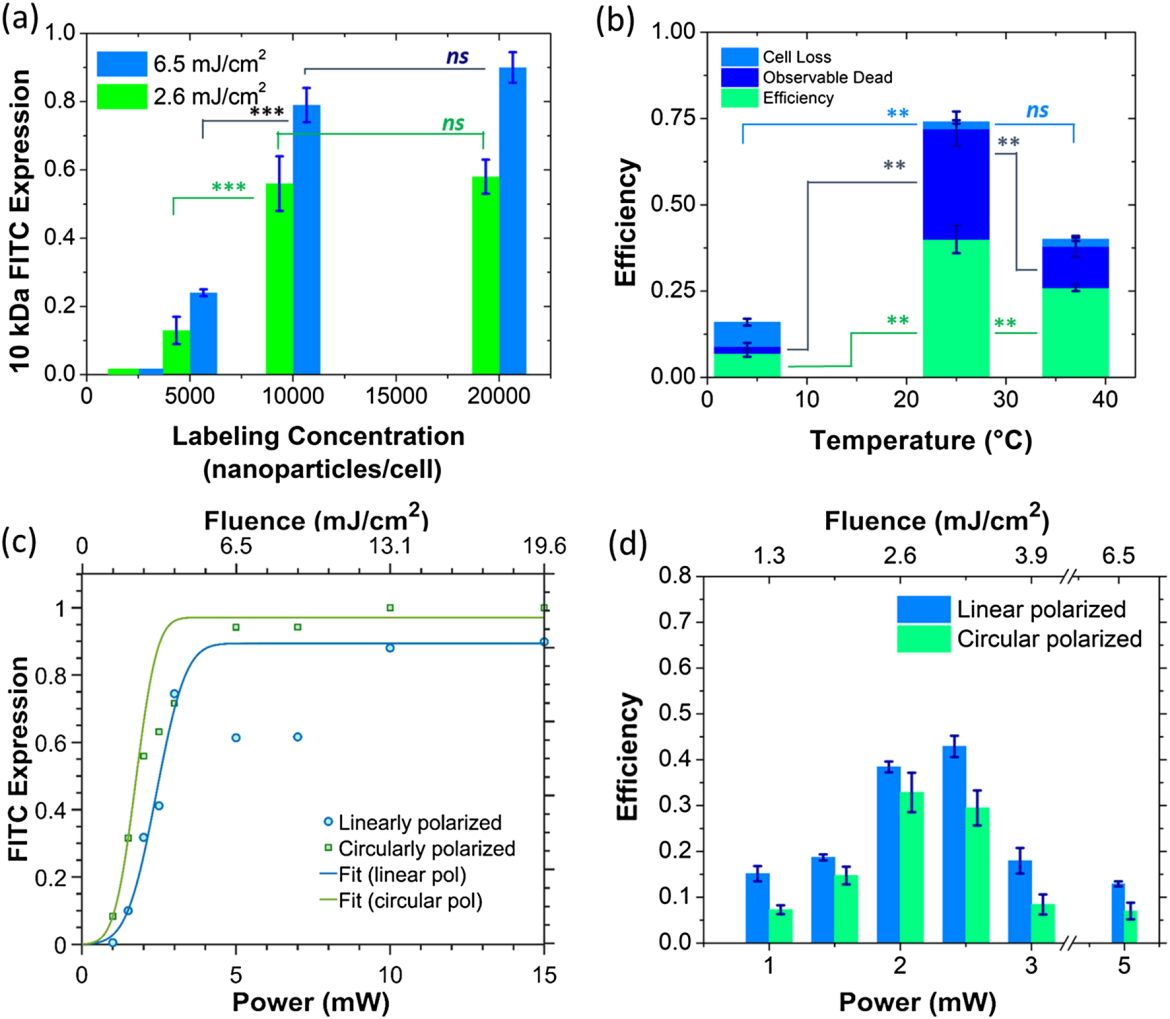
Localization effects on the efficiency of optoporation using PLN. (**a**) The effect of labeling concentration on the PLN optoporation. Measurements of initial intake of 10 kDA FITC-Dextran into the cells as measured 5 min after irradiation with two-photon fluorescence imaging without washing in a new set of experiments (n = 3). (**b**) The effect of temperature during nanoparticle labeling on the PLN efficiency. n = 13 at 25 °C, n = 5 for 4 °C and 37 °C. (**c**) The effect of laser polarization on initial intake of FITC-Dextran over different input fluences in 2 sets of new experiments. Data from these runs are plotted and fit to **Eq. **1. (**d**) The effect of laser polarization on PLN efficiency. Labeled cells were scanned 30 times using linearly and circularly polarized laser pulses over a pulse fluence range of 1 to 6.5 mJ/cm^2^ (n ≥ 3). *p* > 0.01 (*not significant, ns*), *p* < 0.01 (*), *p* < 0.001(**), *p* < 0.0001(***) for two-tailed *t*-test.

Labeling concentration is expected to be an important parameter affecting optoporation efficiency. Nanoparticle concentrations directly influence the size of clusters on the cell surface. To this end, we measured the number of cells incurring FITC-Dextran uptake for cellular labeling concentrations ranging from 2,500 to 20,000 gold bioconjugates per cell using two-photon microscopy (Fig. 3a). PLN was performed with 30 scans of 2.6 and 6.5 mJ/cm^2^ laser pulse fluences and 10 kDa FITC-Dextran was the fluorescent probe. At both fluences, increasing the labeling concentration beyond 10,000 particles per cell produced no significant increase in the intake of fluorescent probe (*p* > 0.1), while halving the concentration to 5,000 particles per cell more than halved the FITC-Dextran intake levels. Further reducing the labeling concentration below this ‘critical’ value to 2,500 particles per cell resulted in no optoporation at either fluence level. The results align well with our hypothesis that increasing labeling concentrations up to a certain value increases the overall formation probability of the larger size clusters, driving up the rate of cells undergoing membrane dysfunction. Reducing the concentration below a critical value, likewise, cannot produce sufficiently large clusters to cause optoporation.

#### Role of EGFR trafficking and nanoparticle endocytosis

Cells have been shown to transport and cluster nanoparticles as part of the EGFR membrane trafficking process^13,47^. Interestingly, temperature affects the speed at which EGFR endocytic trafficking occurs, and controlling temperature has been shown to arrest the EGFR mediated endocytosis process at different stages^13,47,50–52^. For example, labeling at 4 °C inhibits the endocytosis process, retarding EGFR dimerization and clustering. At 25 °C, intracellular trafficking is blocked, with dimerized EGFR clustering in clathrin-coated pits on the interior of the cellular membrane and as early endosomes. If these processes are responsible for the particle clustering and corresponding synergistic effects, a sharp drop in optoporation efficiency would be expected when labeling and optoporating at 4 °C as compared to 25 °C.

We measured the optoporation efficiency at varying labeling temperatures while maintaining a labeling concentration of 20,000 gold bio-conjugates per cell. As expected, Fig. 3b shows that labeling at 25 °C provided the highest PLN efficiency (40% ± 4%), while labeling at 37 °C and 4 °C both showed reduced efficiencies (26% ± 1% and 7% ± 1%, respectively) in a statistically significant manner. A similar trend was found with observable death after PLN. The significant reduction in FITC-Dextran uptake levels compared to 25 °C clearly shows that EGFR based endocytic trafficking was driving the cluster formation on the cellular membrane and facilitating the membrane photomodification during the optoporation process.

The existence of optoporation at 4 °C, albeit at reduced rates, may be explained by the heterogeneity of EGFR along the cell membrane that has been observed in cell types over-expressing the receptor^49^, which enables close interparticle interactions to occur even at 4 °C.

The reduced optoporation rates at 37 °C can also be explained by the increased particle internalization. At high temperatures, it has been shown that clathrin-coated pits are cleaved and result in highly internalized endosomes containing particle clusters when labeling at 37 °C^13,47^. Experiments have previously demonstrated that particles localized in the cytosol require four times more incident energy to achieve similar levels of cellular death as membrane targeted nanoparticles^53^, which explains both the lower efficiency and reduced death rate at 37 °C. The results in this section further highlight the spatial confinement of photo-activated processes involved in fs-PLN membrane optoporation.

#### Effect of laser polarization and cluster geometry

To gain insight on the geometric features of the clusters forming on the cell surface, we treated the labeled cells with linear and circularly polarized laser pulses and measured the properties of the 10 kDa FITC-Dextran uptake (Fig. 3c). Fitting the fluorescent probe uptake data to Eq. 1 showed a small but insignificant decrease in PLN optoporation threshold when irradiating labeled cells with circular instead of linearly polarized light. The percentage of optoporated cells meanwhile plateaued at a higher rate of 98% for circularly polarized light compared to linear polarized light. The percentage of cells exhibiting necrosis also increased with circular polarization at higher pulse fluences of 19.5 mJ/cm^2^ (Supplementary Fig. 2). Approximately 1–2% of cells remained viable when exposed to circular-polarized laser pulses, markedly lower than the 5% that remained viable after linearly polarized irradiation. This reduction in viability could point to circular polarization being preferred in applications such as cancer treatment. Comparing optoporation efficiencies, we found circular polarization to be less efficient compared linearly polarized light, as seen in Fig. 3d. However, the results were statistically insignificant (*p* > 0.01) over most of the fluence range. These results indicate that circularly polarized light is able to activate clusters that are unfavorably oriented for enhancement with linearly polarized light, but not in a manner that improves the optoporation efficiency in a statistically significant manner.

### Characterization of the effect of ROS generation

**Figure 4:**
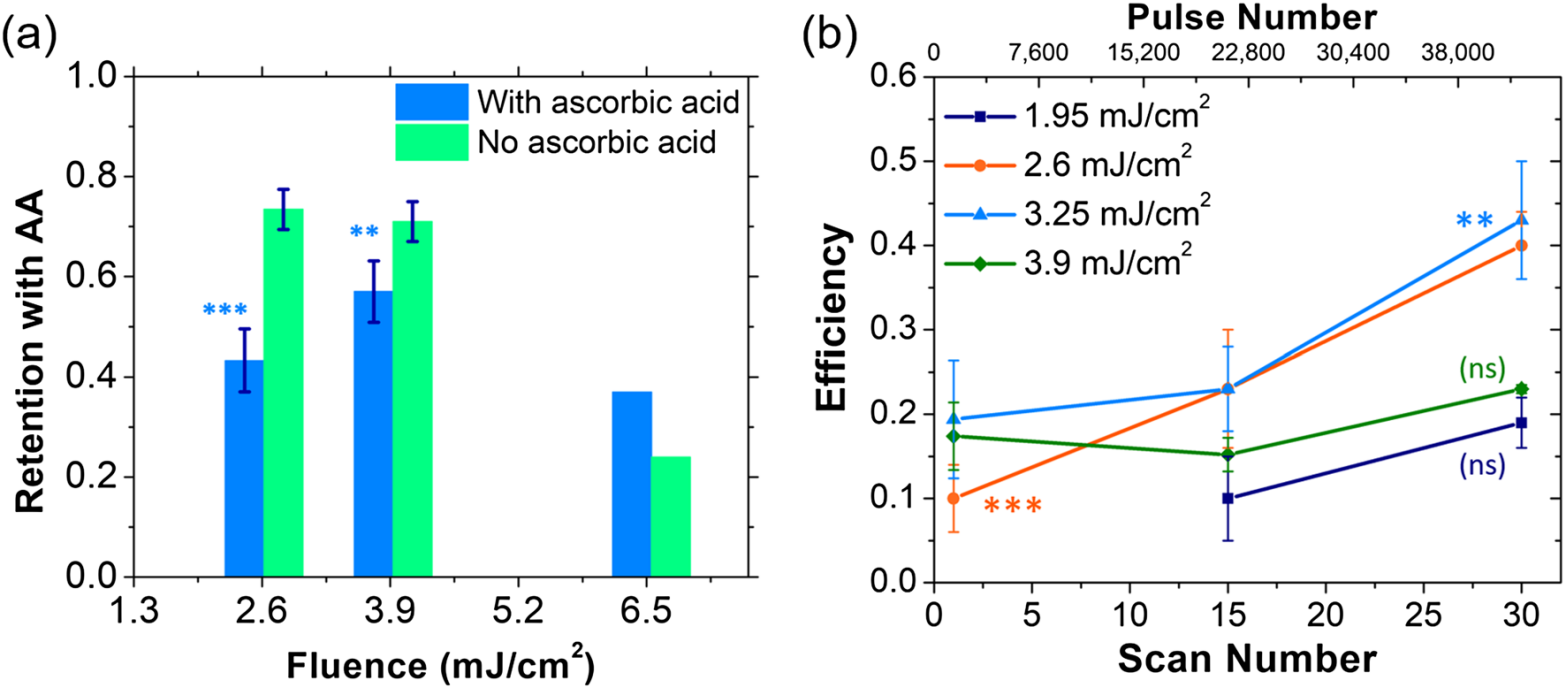
Effect of ROS generation on optoporation threshold and efficiency studied using ROS scavenging by ascorbic acid. (**a**) Reduction in cellular retention rates due to the ROS scavenging by ascorbic acid. We believe ascorbic acid acts to reduce the ROS generated at particle sites, impeding lipid peroxidation and thus pore formation (n = 3). (**b**) The optoporation efficiency for different scanning numbers of the whole FOV and the corresponding total number of pulses per spot. The increase in the number of laser pulse per location occurred while keeping consecutive pulse overlap per single line-scan constant at 378 pulses (over 4.7 µs), and total number of pulses per FOV constant at ~ 1,420 pulses (~ 3.8 line-scans over the same spot). There was a 330 ms interval (time for a single scan of the FOV) before the laser returned to the same location again. The error bars represent the standard error of the mean. *p* > 0.01 (*not significant, ns*), *p* < 0.01(*), *p* < 0.001 (**), *p* < 0.0001(***) for one-way ANOVA.

In conventional femtosecond laser ablation, tightly focused laser pulses with intensities below what is needed to reach the critical electron density, have been shown to affect membrane integrity in cells via ROS formation, produced by the interaction of low density plasma with biomolecules^4,8,40^. Our low optoporation fluences, therefore, led us to hypothesize ROS as a possible source of nanoparticle-mediated fs-PLN photomodification process. To test this hypothesis, we studied the PLN optoporation rates in cells incubated with high concentrations (5 mM) of ascorbic acid (Vitamin C), a well-known anti-oxidant and effective ROS scavenger^54,55^. We found that ascorbic acid produced a significant drop in the number of cells taking in and retaining FITC-Dextran at pulse fluences 2.6 and 3.9 mJ/cm^2^ (Fig. 4a). With ascorbic acid actively quenching ROS formed during PLN, such a decrease in optoporation rate would be expected for a ROS-induced mechanism.

To further understand the effect of ROS formation, we also studied cumulative effects of long-lived ROS by varying number of laser scans thus the total number of overlapping pulses. In one full FOV scan period of 330 ms, an average of 1,420 laser pulses overlap in a single spot. When we increased the number of full FOV scans from 1 to 15 to 30 (Fig. 4b), we found a small but statistically significant improvement in PLN optoporation efficiency with increasing scan numbers at low pulse fluences (2.6 and 3.9 mJ/cm^2^). Such an increase in the optoporation efficiency further supports the hypothesis for cumulative laser-induced ROS generation scan-over-scan. ROS intermediaries in membrane peroxidation have been shown to have lifetimes in the order of seconds^56–58^. During 30 full FOV scans, accumulation and seeding over successive scans can potentially overwhelm local anti-oxidative systems of the cell by additively introducing ROS at the cluster sites, leading to localized pore formation and membrane dysfunction^40^.

Our ROS-based hypothesis is further supported by pore size measurements using FITC Dextran and small interfering RNA to mimic transfection (Supplementary Note 2). A pore radius of 18 ± 5 nm has been observed for both cases using fluorescence rise time measurements (Supplementary Fig. 3). Comparable pore radii and a similar damage mechanism were observed in optoporation experiments using tightly focused NIR, femtosecond laser pulses at high repetition rates in cells sans nanoparticles^4,59^.

Similar ROS-based optoporation has been demonstrated using cold atmospheric plasma, where a non-equilibrium plasma with hot electrons and cold ions has been shown to induce pore formation in vesicles^60–63^, artificial lipid bilayers^64^, and through molecular dynamics simulations^65,66^. Lipid peroxidation was determined to be the mechanism of pore formation^64^. We hypothesize our optoporation mechanism follows similar pathways, where nanoparticle clusters, upon excitation by femtosecond pulses, emit seed electrons resulting in free radical synthesis and subsequent lipid peroxidation generated via PLN. Lipid peroxidation has been shown to cause structural disorganization of the membrane, loss of membrane protein function, and increase in membrane fluidity^64,65,67^. While our results add to the overwhelming evidence of a ROS-based optoporation mechanism, further work is needed to deterministically confirm the effect of ROS in such a setting.

### Characterization of the PLN membrane permeabilization mechanism

Having gained valuable insight into the effects of nanoparticle clusters and possible role of ROS-formation on the photomodification process at ultralow laser pulse fluences, we shifted our focus to further studying the underlying mechanisms, including heating, photoionization, and ROS-induced membrane photodisruption mechanisms.

To analyze these effects, we first need to determine the expected enhancements in absorption and Poynting vectors in the near-field of the particle clusters. These enhancements depend on how the nanoparticles are distributed in the clusters and how tightly they are packed^68–75^. The cluster packing factor (*s/d*), given by the inter-particle distance (*s*) relative to the particle diameter (*d)*, is a key determinant of the extent of enhancement^69^. Crucially, the extent of plasmon red-shift and/or broadening in the absorption/extinction spectra provide valuable information towards determining the number and packing factor of particles^69–71,76,77^. Different clustering geometries may also result in the emergence of a second axis of symmetry and a clearly discernable split in the plasmon band^69,72,73,76^. The measured spectrum of our labeled cells in suspension (Supplementary Fig. 1a) only shows a small red shift (~4 nm) and no peak splitting compared to the nanoparticles in suspension, indicating that, on average, the clusters were not tightly packed. Based on the previous studies, the resonance peaks red-shift^68,70,71^and enhancements increase^69,72^ with decreasing packing factor. We take *s*/*d* = 0.8 as the baseline for our calculations, since it has been shown to produce our observed 4 nm red shift in the resonance peak , in 42 and 50 nm particles^68,70,71^.

Previous scanning electron microscopy studies have shown that EGFR–conjugated nanoparticles in cells form linear or two-dimensional (2D) clusters, with the nanoparticles following the endosome or cell membrane profile^13,47^. Our polarization independent optoporation results were indicative of a relatively symmetric clustering configuration. Considering these factors, we chose a 4-particle cluster made up of 47 nm particles arranged in a 2D square pattern with a packing factor (*s/d*) of 0.8 to represent an average cluster we might encounter in our experiments. The enclosed 2D geometry rather than a linear one also accounts for any thermal confinement in the interstitial region between the particles, representing potentially an extreme case for particle heating. Finite volume solutions to Maxwell’s equation via a finite difference frequency domain (FTFD) scheme was used to calculate the absorption cross-section and the near-field enhancements for the cluster^78,79^. We calculated an absorption cross-section of 124 nm^2^ for a single 47 nm gold nanoparticle excited at 760 nm and 514 nm^2^ for the 4-partile cluster separated by 37.6 nm (*s/d* = 0.8), a 4.2-fold increase over a single particle. Our simulations also showed a maximum 1.3-fold increase in Poynting vector enhancement for the 4-particle cluster (*s/d* = 0.8) as compared to a single 47 nm nanoparticle (Supplementary Fig. 4). Interestingly, the results showed only 10% increase in the Poynting vector enhancement (with a maximum enhancement of 10.0) and only 5% increase in the absorption cross-Sect. (542 nm^2^) for a denser packing density of *s/d* = 0.6.

**Figure 5:**
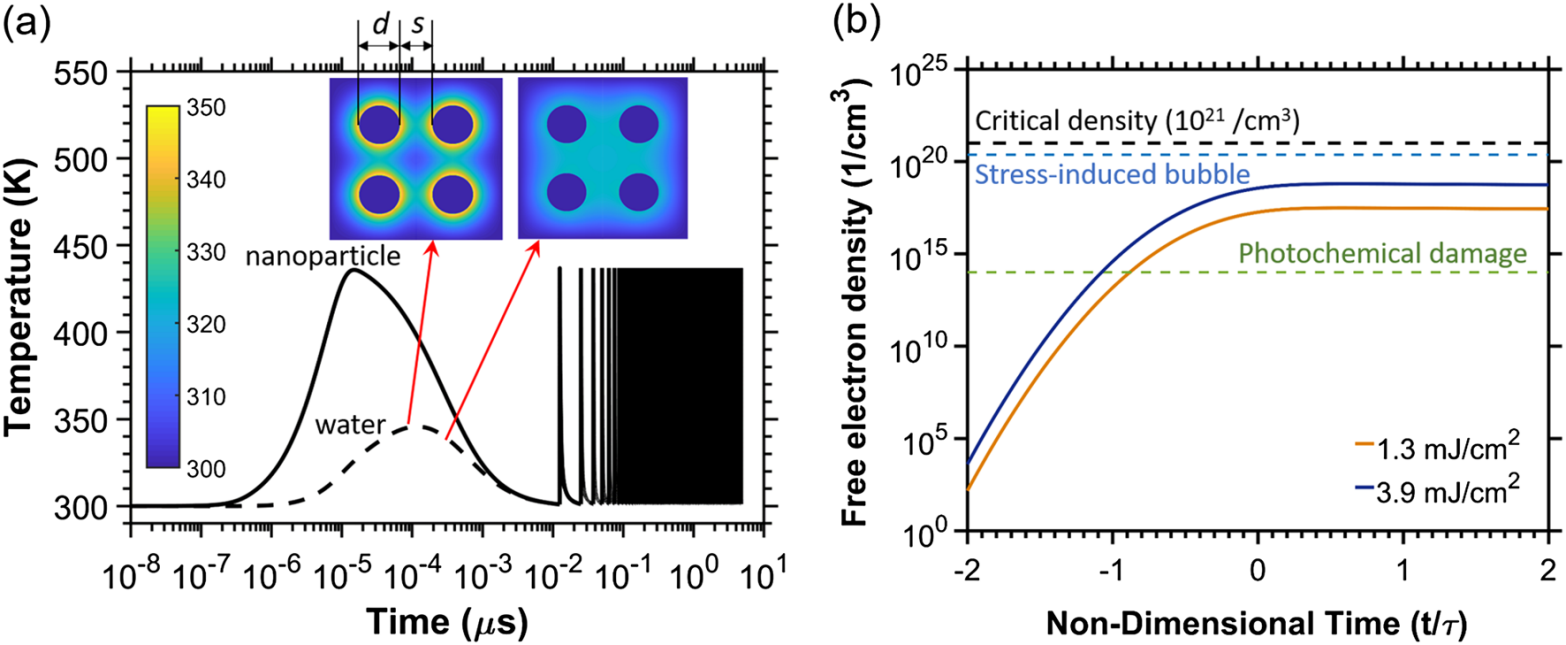
Thermal and photoionization simulations. (**a**) The temperature dynamics of a cluster with 4 gold nanoparticles (*d* = 47 nm) with packing factor of *s/d* = 0.8 and surrounding water at an average pulse fluence of 3.9 mJ/cm^2^ (pulse energy of 37.5 pJ) over the exposure time of a spot in the FOV during a single line-scan, which comprises of 378 consecutive laser pulses. For conservative calculations, we assumed the particles are located at the laser’s focal center and experience the highest local fluences (the peak fluence), equal to twice the average input fluence. We used the absorption coefficient calculated for the 4-particle cluster with packing factor of *s/d* = 0.8, excited with 760 nm laser light from the FTFD simulations. The solid line represents gold lattice temperature. The dashed line represents the temperature evolution of water near the particle interface where the water temperature reaches the highest values. Insets show the temperature map of water surrounding the particle at different time points, with particle temperatures removed for clarity. The peak water temperatures quickly decayed to room temperature before the arrival of the next laser pulse. (**b**) Curves showing the free-electron generation in water adjacent to the surface of a particle in our cluster over time, accounting for the estimated maximum intensity enhancement of 9 times at the particle surface and free-electrons photoemitted from the particles. In these calculations, we again assume that the particles experience the peak fluences (twice the average input fluence locally) at the laser’s focal center. Here, τ refers to the pulse width of 270 fs.

To understand the role of heating in our optoporation mechanism, we modeled the temperature rise of the particle and surrounding water in the cluster when interacting with 378 consecutive pulses of incident laser light at 80 MHz and 270 fs pulse width. While during full FOV scan, there are 1,420 overlapping laser pulses in total, only 384 pulses arrive consecutively during the line-scan which can lead to heat accumulation. We assumed that the particles interacted with the highest local fluence possible within a laser beam, which is the peak fluences located at the beam center, where the local fluence is equal to twice the average input pulse fluence. A two temperature model solved via a 2D finite volume scheme was used^11^. The thermal simulation results show that at our observable threshold pulse fluence of 1.3 mJ/cm^2^ (pulse energy of 12.5 pJ), the maximum transient temperature increase in water reached approximately 18 K near the particle surface, and quickly decayed to room temperature by diffusion before the arrival of the next pulse 12.5 ns later (Fig. 5a). A similar trend was observed at a higher pulse fluence of 3.9 mJ/cm^2^ (37.5 pJ), which produced a maximum transient temperature increase of 50 K in the water and 140 K in the gold lattice again with almost no temperature accumulation over 378 pulses (Fig. 5a).

We performed additional thermal calculations for the full range of fluences used in our experiments to examine if temperature accumulation can potentially produce bubble nucleation. The liquid phase of water becomes unstable beyond 80 – 90% of the critical temperature of water (*T*_*cr*_ = 647 K), and has been indicated as the threshold for bubble formation in nanoparticles of similar sizes^80–82^. Note that the thresholds mentioned here refer to bubble nucleation through direct particle heating and heat transfer to the surrounding water, and not the direct plasma heating of the water due to electron thermalization which occurs with much higher levels of stress confinement. The calculations presented in Supplementary Fig. 5 showed that the surrounding water could not reach temperatures high enough for bubble nucleation at any of the operating fluences. The particle lattice temperatures were also sufficiently low with no effect on particle morphology due to fragmentation or melting. These results also showed that even at the highest pulse fluences (9.1 mJ/cm^2^, 87.5 pJ), heat accumulation did not exist anywhere in the field including the cluster center, where the heat diffusion is confined (Supplementary Fig. 5). Our analysis, therefore, indicates a diminished role of thermal effects in our optoporation process on average.

With thermal effects addressed, we simulated free-electron generation in the near-field of particles to assess if particle photoemission and free-electron generation in the surrounding liquid media might drive optoporation (Fig. 5b). A first-order model was used to describe the combined effects of particle photoemission and photoionization of the surrounding media^20,39,83,84^,

2 $$\frac{\partial \rho }{{\partial t}} = - \nabla \cdot J_{n} + \eta_{photo} + \eta_{casc} \rho_{c} - \eta_{diff} \rho_{c} - \eta_{rec} \rho_{c}^{2}$$

The first three terms on the right side of the equation represent free-electron generation rates associated with particle emission ($\nabla \cdot J_{n}$), multiphoton ionization in water ($\eta_{photo}$), and cascade ionization in water ($\eta_{casc} \rho_{c}$), and while the last two terms represent the diffusive ($\eta_{diff} \rho_{c}$) and recombinatory ($\eta_{rec} \rho_{c}^{2}$ ) losses. Photoemission rates were calculated using the generalized Fowler-DuBridge theory^85^, which has been used to successfully describe a combination of thermionic and multiphoton assisted electron emission in thin films^85,86^. Free-electron generation in water (all last 4 terms on the right side of the Eq. 2) was modeled using a combined Keldysh-Drude model^87,88^. The non-uniform near-field Poynting vector enhancement (Supplementary Fig. 5) arising from the particles was introduced into the photocurrent density equations through the laser intensity source term. Again, we assumed the particles were located at the laser’s focal center, and experience twice the average pulse fluence.

As we solved each term of the rate equation, the photocurrent from the particle was used to estimate the threshold for particle ablation. The photocurrent generated breaks the charge quasi-neutrality in the particle resulting in an electric field on the particle surface, which can be determined using Gauss’s law. When this electric field reaches a threshold value (27.6 V/nm for gold^86^), bonds are broken and the surface disintegrates via a Coulomb explosion process^86,89^, resulting in particle ablation.

To estimate the thresholds for plasma-induced bubble formations in water, we simulated the temporal evolution of the free-electron density in water right next to the particles in the cluster after irradiation using Eq. (2), considering the photoemitted electrons from the particle as described above. Multiphoton and cascade ionizations in water, and the diffusion and recombination losses from our volume in consideration similar to Vogel et al*.*^39^, where water is treated as a dielectric medium with an ionization potential of 6.5 eV.

We do not expect the free-electrons generated from neighboring particles in the cluster to interact with each other over the duration of the pulse since plasma diffusion is quite slow, traveling ~ 10 nm over the duration of one laser pulse^90,91^. As such, free-electron generation simulations are performed for a single particle, with field enhancements accounting for multi-particle interactions. The photo-emitted electrons from gold are assumed to participate in the cascade ionization in water over a volume defined by their diffusion length for a given time.

The critical electron density above which the dense plasma becomes strongly reflective and absorbing, was set at 1.9 × 10^21^ electrons/cm^3^ for our simulations^39^. It is important to note that recent studies define the optical breakdown terminology to be associated with the threshold of experimentally detectable bubble formation, namely stress-induced bubble formation^92^. Here, we refrain using the terminology of optical breakdown to avoid confusion with this definition and its traditional definition associated with the critical density formation. Instead, we term this threshold as the “critical electron density-induced bubble formation threshold.” Further details of our full simulation are given in the Supplementary Note 3.

**Figure 6:**
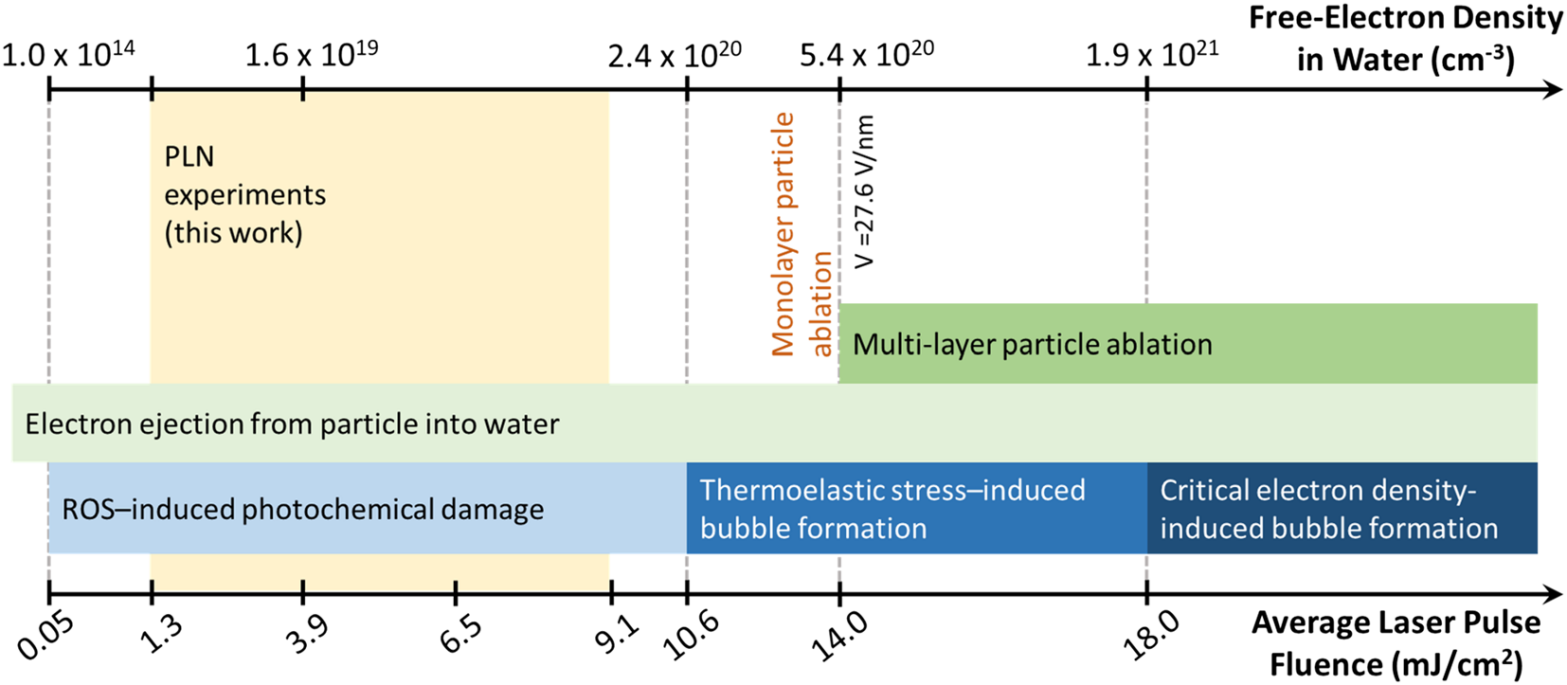
Summary of photoionization simulations, describing the various regimes of PLN driven by a 760 nm, 270 fs laser pulse interacting with a 47 nm gold nanoparticle in a 4-particle cluster with a packing factor *s*/*d* of 0.8. Fluences used in the simulations assume the particles are located at the focal center, experiencing the highest local fluences possible, namely the peak fluences (equivalent to the twice the average laser pulse fluence). Initiation thresholds for different phenomena are indicated along the vertical dashed lines. The model calculates the free electrons generated from a single particle experiencing enhanced fields from the particle cluster. Since electron diffusion is very slow, we assume that the free electrons from neighboring particles in the cluster do not interact. Particle emission seeds both ROS formation and multiphoton ionization in water. At the pulse fluence threshold of 10.6 mJ/cm^2^, we predict enough electrons would be generated in the low plasma density regime to initiate thermoelastic stress-induced bubbles (defined as the “optical breakdown threshold” in Linz et al*.*^92^). With the increasing number of free-electrons, the E-field on the particle can become strong enough to result in Coulomb explosion and monolayer ablation at 14 mJ/cm^2^. Further increase in laser pulse fluence produces critical free-electron density at 18 mJ/cm^2^. Particle shape change and resulting near-field effects are not modeled in conjunction with the free-electron generation. Full particle ablation is not modeled as plasma shielding effects after reaching critical electron density and space-charge effects due to ion ejection are not included in calculations.

The PLN photoionization diagram in Fig. 6 reveals which mechanism is prominent in distinct regions of average laser pulse fluence. In these calculations, we again assumed that the nanoparticles are exposed to the peak fluences (twice the indicated average pulse fluence). Therefore, the thresholds estimated here present the most conservative calculations with the lowest possible pulse fluence thresholds. At our optoporation fluences in the range of 1.3 to 9.1 mJ/cm^2^, we found that the free-electron density was at least an order of magnitude lower than the critical density, but sufficient enough to induce ROS-based chemical cell damage leading to optoporation^1,39^. To calculate the threshold for stress-induced bubble formation, we assume that stress confinement in water mimics that of plain water as described by Vogel et al.^39^. For our NA, a temperature rise of approximately 430 K would produce stress-induced bubble formation^93^. At 10.6 mJ/cm^2^, sufficient free-electron density is generated, which when thermalized, should initiate thermoelastic stress-induced bubble formation. Further increase in fluences results in strong E-fields, generated from breaking the quasi-charge neutrality in the particle due electron ejection. While not sufficient for complete particle ablation, these E-fields become strong enough for monolayer ablation of nanoparticles, starting at 14 mJ/cm^2^. The estimated thresholds (18 mJ/cm^2^) for critical electron density in water are even higher than the monolayer ablation threshold.

Reducing the packing factor to *s/d* = 0.6, which further increases the enhancement, did not produce any significant change in the expected mechanism at our operating fluences, although the threshold of stress-induced cavitation bubble formation did move closer to our highest operating fluences (Supplementary Fig. 6). It is likely, therefore, that at our operating fluences, low-density plasma drives the optoporation process via ‘gentle’ chemical effects like ROS generation. At higher fluences, we find that other ablation effects might come into play including stress-induced bubble formation, monolayer ablation of particles, and critical electron density-induced bubble formation that may be considered more “violent”. It is interesting to note that the optoporation results we obtained at or near higher fluences (> 8 mJ/cm^2^) resulted in increased cell death.

**Figure 7:**
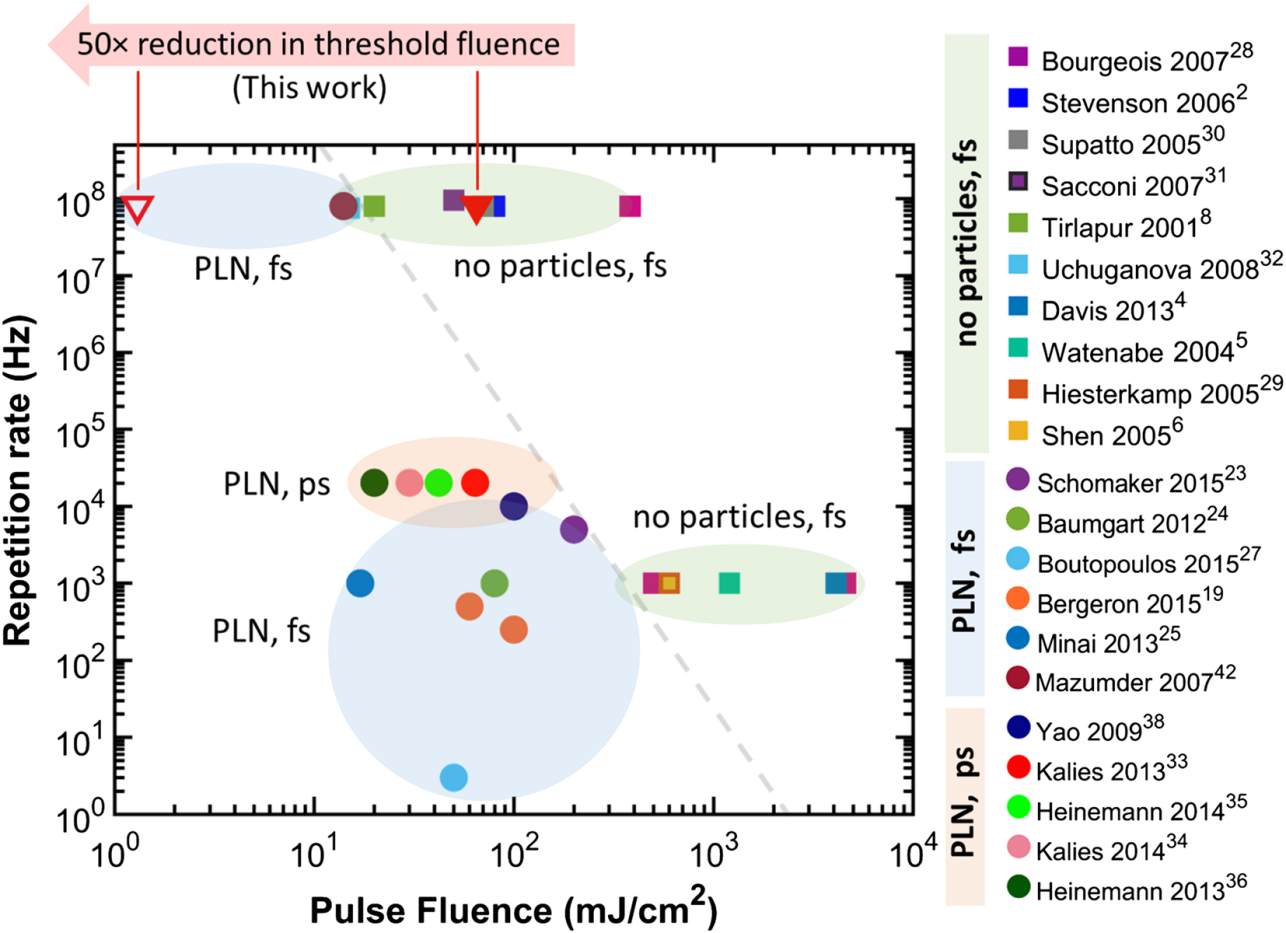
Literature summary of laser pulse fluences used in different photomodification studies in different regimes of laser repetition rates. The bubbles highlight the method used for photomodification; with picosecond (ps) and femtosecond (fs) laser pulses and with and without the aid of nanoparticles. Distinct regions are visible for each method. The red triangles represent our results in this study, with solid triangle representing the fs-laser irradiation of cells without nanoparticles. This plot shows a few general observations: (1) Moving to high repetition rates reduces the thresholds for conventional fs laser surgery by up to 2 orders of magnitude. (2) Femtosecond laser assisted PLN with gold nanospheres produces a 10–100 times reduction in threshold with respect to conventional fs laser surgery at the corresponding repetition rates. (3) Consequently, up to 2 orders of magnitude threshold reduction occurs for PLN at MHz repetition rates compared to kHz operation. A summary of all operating parameters is tabulated in Supplementary Tables 1 and 2.

In all, Fig. 7 shows where PLN lies on a map of the pulse fluences versus repetition rates for different photomodification modalities explored in literature using femtosecond and picosecond laser pulses. The combined use of high repetition rate lasers and nanoparticles produces up to two orders of magnitude reduction in thresholds compared to the using either method in isolation.

We can conclude that our ability to induce localized membrane disruption at such low fluences stems from nanoparticle clustering on the particle membrane, acting as local concentrators of free-electron generation. Based on the measured spectrum shift and estimated cluster densities, FTFD calculations indicate that the particle clusters in our experiments likely produce field enhancements sufficient to generate free-electron emission. The free electrons emitted from the particles contribute to the photoionization mechanism of water. At high repetition rates these two mechanisms can generate and accumulate a high concentration of ROS in their vicinity that overwhelm the cell’s repair mechanisms at the site and cause optoporation. Recent studies by Ma et al.^44^ and Schomaker et al.^23^, have similarly pointed to the important role particle clustering plays in the membrane optoporation process. Considering how important the binding site dynamics are at the cellular level, it is crucial to design the PLN systems to the specific cell type and accounting for effects like clustering and particle endocytosis.

## Conclusions

This paper describes our experimental and analytical efforts to study the fs-PLN photomodification mechanism of cellular membranes in live cells at ultra-low pulse fluences using high repetition rate, 270 fs laser pulses. By using off-resonant excitation conditions, we suppressed particle heating while exploiting photo-chemical processes to affect efficient membrane permeabilization in triple-negative human breast cancer cells. We revealed that fs-PLN photomodification at a repetition rate of 80 MHz is based on the formation and accumulation of long-lived ROS, reducing the optoporation threshold by one-to-two orders of magnitude as compared to that at kHz repetition rates.

We also showed that optoporation efficiency depends on labeling concentration, which affects the density of particles on the cellular membrane, as well as on the EGFR endocytic trafficking cycle, which affects particle localization and clustering. Oxidative stress experiments in the presence of ascorbic acid pointed to ROS formation as the dominant photomodification mechanism of fs-PLN at ultra-low pulse fluences in the 80 MHz regime. A first order model simulating particle emission and water photoionization further corroborated a low-density plasma-based chemical damage model. Light-particle interaction at off-resonant wavelengths is dominated by field enhancement. At our operating fluences, the femtosecond laser pulses result in photoactivated processes that produce low density plasma from free electrons emitted from the particles and photoionization of the surrounding water. Clusters act to localize and additively increase the free-electron concentrations. The emitted electrons provide the seed for the formation of free radicals in the surrounding water, leading to optoporation by photo-chemical processes.

In conclusion, an ultra-low fluence PLN process using high repetition rate, femtosecond laser pulses, provides an attractive phototherapeutic alternative for biomedical applications. The nanoparticle clusters acting as “nano-lenses”, can permit nano-scale subcellular processing over large regions of cells without the need for precise laser focusing at the target of interest. This property thus makes the PLN technique ideal for in vivo clinical use in the large-scale phototherapy of dysplasia and tumors. Furthermore, for a given laser pulse fluence, the one- to two-orders of magnitude threshold reduction compared to other PLN methods at low repetition rates, can potentially increase photomodification depths by ~ 4–5 scattering lengths (according to Beer’s Law; e^4^ ~ 55) in non-invasive treatments of bulk tissues. With the currently available high repetition rate (< 50 MHz), high average power fiber lasers (up to 100 W), PLN can now provide the next generation surgery method with high selectivity and precision in clinically acceptable timescales.

## Materials and methods

### Multiphoton imaging and plasmonic laser nanosurgery system

Imaging, characterization, and optoporation of monolayer cellular constructs were performed using our home-built upright multiphoton microscope system^94^. A femtosecond Ti:sapphire laser system (Mai Tai; Spectra Physics) delivered linearly polarized, 760 nm wavelength laser pulses at an 80 MHz repetition rate. Two attenuators consisting of a half-wave plate (HWP) and a polarizing cube beam-splitter were used to control the laser power. One HWP was adjusted by a computer-controlled actuator providing 0.01º angular resolution for fine power control. When necessary, an additional reflective neutral density filter (ND = 10) was placed in the excitation path. For the delivery of circularly polarized light, a quarter-wave plate was placed in the excitation path such that the optic axis of the plate was at a 45º angle to the incident laser light. Laser pulses were raster scanned with a saw-tooth pattern onto the back aperture of a water-dipping objective lens (0.95 NA, 20 × ; Olympus XLUMPlanFI) using a two-axis galvanometric scanning mirror system (6215H; Cambridge Technologies). The power transmission through the objective was measured at 33% by finding the ratio of energy before and after the objective. At the sample plane, the pulse duration measured via autocorrelation of the focused excitation beam within a fluorescein sample was found to be 270 ± 10 fs, and the two-photon lateral and axial FWHM resolutions were 460 ± 60 nm and 1,760 ± 130 nm, respectively. The corresponding 1/e^2^ intensity widths were 1.1 ± 0.2 μm and 4.1 ± 0.3 μm in the lateral and axial directions, respectively. Emitted light was epi-collected, reflected by a cold mirror (400–700 nm reflectance, HT-1.00; CVI Laser), passed through a laser filter (BG-38, Low pass < 700 nm; Schott), and detected with a cooled GaAsP photomultiplier tube (H7422-40; Hamamatsu). The sample was mounted onto an automated x–y–z translational stage (NanoMax; ThorLabs).

### Molecular targeting protocols

To study the in vitro optoporation of cancer cells, the cell membranes of MDA-MB-468 triple-negative human epithelial breast cancer cells were labeled with gold nanoparticles. Labeling was achieved by molecularly targeting the epidermal growth factor receptor (EGFR), which is an important biomarker for carcinogenesis. The cellular membranes of these cancer cells express ~ 2 × 10^6^ EGF binding sites per cell^95,96^, which is at least one order of magnitude larger than that of noncancerous cells having 0.4 × 10^5^ to 1 × 10^5^ EGF binding sites per cell^97^. Thus, EGFR might provide a molecularly specific moiety to target cancer cells via functionalized gold nanoparticles.

Gold nanospheres were synthesized using the Turkevich method^98–100^, producing spheroidal particles measuring 54 ± 6 nm, with an ellipticity of 1.24 ± 0.13 (‘equivalent volume’ sphere diameter of *d* = 47 ± 5 nm). The particle surface was functionalized with αEGFR antibodies via direct surface charge based physisorption^101,102^. To prevent antibody desorption from the particle surface during cellular labeling, we maintained serum-free conditions throughout the PLN protocol. To maintain cellular adherence in serum-free conditions and provide a platform for simultaneous imaging and optoporation in a monolayer construct, cells were attached to glass cover slips functionalized with αEGFR using a modified protocol implemented by Maraldo et al*.*^103^. The nanoparticle concentration in the labeling solution was titrated based on the cell count in each petri dish to obtain a desired seed concentration per cell. In general, cells were labeled at 25 ± 2 °C for 20 min using an initial concentration of 2 × 10^4^ gold nanoparticles per cell.

#### Bioconjugate synthesis and characterization:

To prevent competitive binding of citrate ions with antibodies, 2 mL of 56.5 pM gold nanoparticles were centrifuged at 1,500G for 30 min and re-suspended in 1 mL of 20 mM sodium HEPES (4-(2-hydroxyethyl)-1-piperazineethanesulfonic acid) buffer (pH 7.50–7.53; Sigma) to a final molar concentration of 113 pM. The gold nanoparticle solution was mixed 1:1 (v/v) with 103 pM Human αEGFR Monoclonal Antibodies (clone 225, 1.93 mg/mL; Sigma) in 20 mM sodium HEPES Buffer. The solution was allowed to interact for 45 min before stabilization with 400 µL of 15 µM high molecular weight polyethylene glycol (PEG-4000; Fluka). Subsequent centrifugation at 350G for 20 min and 800G for 45 min removed nanoparticles aggregates and unbound antibodies respectively. Two hundred and fifty pico-molar of the bioconjugate pellet was re-suspended in Dulbecco’s Phosphate Buffered Saline (DPBS) buffer without Ca^2+^ and Mg^2+^ (Sigma) and stored at room temperature. The bioconjugate spectrum was measured to have an absorption peak centered at 536 nm with 80 nm FWHM (Supplementary Fig. 1a). Compared with bare particles, the bioconjugates exhibited a 5 nm plasmon red-shift, which is indicative of a well adsorbed protein layer on the particle surface^104,105^. The broadening of the plasmon band by 10 nm and a 3.3 fold increase in absorbance at 760 nm is also consistent with previous observations in bioconjugate solutions^41,106,107^.

#### Preparation and characterization of monolayer cellular constructs

Cover slips were thoroughly washed using a four-step process: methanol-hydrochloric acid solution (1:1, v/v; Fisher), sulfuric acid (N = 12.1; Fisher), hot sodium hydroxide (pH = 14; Baker Scientific), and boiling in deionized, ultra-filtered (DIUF) H_2_O. After washing, cover slips were air-dried and silanized with 3-aminopropyl-triethoxysilane (0.1%; Sigma) in DIUF-H_2_O at pH 3.0 (adjusted with hydrochloric acid, 0.1 N) and 75 °C for 2 h. The zero-length cross-linker 1-ethyl-3-(3-dimethylaminopropyl)-carbodiimide (2 mM, EDC; Pierce) promoted by sulfo-N-hydroxysuccinimide (5 mM, sulfo-NHS; Pierce) was used to activate the carboxylic group present on αEGFR antibodies 30 min prior to reaction with the amine-functionalized glass cover slip. The molar ratios of EDC and sulfo-NHS to αEGFR antibody used per cover slip were 81:1 and 103:1, respectively. Upon silanization, the cover slips were ultra-sonicated for 5 min in ethanol (200 proof; Fisher), rinsed in DIUF-H_2_O, and air-dried. A well isolator (0.5 mm depth, 9 mm diameter; Invitrogen), unto which stable intermediates and cells were applied, was adhered to the cover slip surface. Covalent coupling of the stable intermediate with the free amine terminals was carried out at room temperature for 5 min. Before cellular seeding, unbound antibodies were flushed with 100 μL DPBS.

MDA-MB-468 cells were maintained by culturing in Dulbecco’s modification of Eagle’s medium (DMEM; Gibco) supplemented with 10% Fetal Bovine Serum (Sigma), 100 U/ml penicillin (Sigma), and 100 g/ml streptomycin (Sigma) at 37 °C in a humidified 5% CO_2_ atmosphere were cleaved from the flasks using a solution of 0.25% w/v trypsin (Gibco) and seeded onto the prepared cover slips at a cellular density of 4 × 10^5^ cells/well. Plates were incubated for 24 h at 37 °C in a humidified, 5% CO_2_ atmosphere before optoporation experiments.

Since nanoparticles brightly luminesce when excited with high peak intensity femtosecond laser light^46,108^, we used multiphoton luminescence (MPL) imaging to characterize cellular labeling. Supplementary Fig. 1b provides a representative MPL image of cells labeled in suspension and assembled as a single layer at the bottom of a petri dish. Bright MPL rings present along the cellular membrane indicated that the EGFRs distributed along the membrane were successfully labeled by αEGFR gold bioconjugates^46,47^. The average measured spectra of labeled cells in suspension Supplementary Fig. 1a gave an expected absorption peak centered at 540 nm, with a bandwidth of 80 nm FWHM awith almost no broadening as compared to bioconjugated nanoparticles (536 nm peak, 80 nm FWHM) and a slightly broadened as compared to bare nanoparticles (531 nm peak, 70 nm FWHM). The measured spectra were utilized to estimate the concentration of nanoparticles bound per cell^109^.

### Optoporation of labeled cells via PLN

Before PLN, we imaged a 150 × 150 μm^2^ FOV of nanoparticle-labeled cells with 760 nm wavelength laser light at a 0.65 mJ/cm^2^ (0.5 mW) average fluence. The 760 nm wavelength was chosen for two reasons: (a) the two-photon cross-section is the highest within the laser parameters available to us^110^ and (b) the ratio of the particle’s near-field scattering to absorption is high, minimizing heating effects during both imaging and PLN. Observation of multiphoton luminescence shows the distribution of labeled nanoparticles along the cellular membrane (Fig. 1a and Supplementary Fig. 1b). The multiphoton luminescence signature of cells labeled after adherence to the coverslip was generally reduced and less uniform than patterns found with cells labeled in suspension. A reduction in the available number of surface receptors onto which the nanoparticles can attach due to our cellular adherence technique, and the “sticky” nature of the functionalized glass cover slip attracts nanoparticles which effectively reduces the number of available nanoparticles for labeling are two potential reasons for this signature change.

To image through the entire cellular volume, we obtained a 14-layer MPL image stack with a 2 μm step size. To perform PLN, we selected the center plane of the cell layer and scanned the laser spot along 512 lines in a saw tooth pattern in the x–y plane across the entire 150 × 150 µm^2^ FOV. One full scan took about 330 ms. Considering the measured 1/e^2^ beam diameter of 1.1 ± 0.2 μm, the dwell time over one laser spot was approximately 4.7 µs, resulting in 378 consecutive pulses to be overlapped per spot after each line-scan and a total of ~ 1,420 pulses after one full FOV scan.

Either FITC-Dextran (10–150 kDa, 25 mg/ml; Sigma) or siRNA mimics (14.9 kDa, p-GUCAUUGCCACCCAGCUACUGG-fl sequence; Dharmacon) were used as fluorescent probes. The probe was added into the extracellular solution 30 min prior to PLN irradiation. Both FITC-Dextran and the siRNA mimics are membrane impermeable macromolecules, and their uptake was utilized to indicate membrane dysfunction resulting from PLN (Fig. 1a-c). To minimize particle internalization from EGFR trafficking^50,111^, labeling was performed at 25 °C, unless otherwise stated, and PLN was completed within 30 min of labeling. This time constraint limited the maximum number of irradiation zones per plate to approximately 10. Each irradiation zone was separated by 500 μm, center-to-center. FITC-Dextran influx into the cytosol was monitored by two-photon fluorescence 5 min after PLN.

### Determination of efficiency of cellular optoporation

Cell viability was probed with calcein red–orange, AM (14 μM; Invitrogen), an intrinsically fluorescent cell-permeant dye that is well-retained by live cells that possess intact plasma membranes, after washing away the extracellular FITC-Dextran and before moving the sample to the fluorescence microscope^112^. Viability imaging was performed using an epifluorescence microscope (BX51; Olympus) 30 min after the cells were irradiated. After irradiation in the multiphoton microscope setup, FITC-Dextran was washed away with DPBS and the number of intracellularly fluorescent cells was counted both 5 and 30 min after irradiation using two-photon fluorescence and epifluorescence imaging, respectively. The filter sets for FITC and tetramethyl rhodamine isothiocyanate (TRITC) were used for epifluorescence imaging to isolate signals from FITC-Dextran (green) and calcein red–orange, AM (red), respectively.

### Monitoring transient influx of FITC-Dextran after optoporation

For transient analysis of FITC-Dextran influx, we measured the average extracellular fluorescence intensity in five 5 × 5 μm^2^ regions containing no cells in two-photon fluorescence images. Within the FOV, we measured the fluorescence levels of 5 unporated cells and 25 cells that incurred FITC-Dextran uptake. Intracellular fluorescence levels were measured within an intercellular region maintaining a 10-pixel margin from extracellular fluorescence. All optoporated cells with FITC-Dextran influx had an associated swelling not seen in cells with intact membranes. At the plane of imaging, an increase in area of approximately 50%, on average, was noted in the intercellular region. The region of interest was increased with time to compensate for intracellular swelling by that amount. To determine the change in fluorescence intensity with time, the data was offset by the initial signal immediately before irradiation as well. All analyses were performed using ImageJ.

## Supplementary information

Supplementary information.

## Data Availability

The datasets generated during and/or analyzed during the current study are available from Adela Ben-Yakar (ben-yakar@mail.utexas.edu) and Daniel Eversole (eversole@gmail.com) on reasonable request.
