# Supplementary material for: Femtosecond Plasmonic Laser Nanosurgery (fs-PLN) mediated by molecularly targeted gold nanospheres at ultra-low pulse fluences
Source: Sci Rep. 2020 Jul 24;10:12387. doi: 10.1038/s41598-020-68512-2 (PMC7382507; doi:10.1038/s41598-020-68512-2)
Supplement: Supplementary file 1 — Supplementary information. [file 41598_2020_68512_MOESM1_ESM.pdf]

Supplementary information

Femtosecond Plasmonic Laser Nanosurgery (fs-PLN) Mediated by Molecularly Targeted Gold Nanospheres at Ultra-Low Pulse Fluences

Daniel Eversole[1,+], Kaushik Subramanian[2,+], Rick K. Harrison[2], Frederic Bourgeois[2], Anil Yuksel[2], and Adela Ben-Yakar[1,2,*]

[1]Biomedical Engineering, The University of Texas at Austin, Austin, TX, 78712, USA

[2]Mechanical Engineering, The University of Texas at Austin, Austin, TX, 78712, USA.

[*]Correspondence and requests for materials should be addressed to A.B. (email: ben-yakar@mail.utexas.edu)

[+]These authors contributed equally to this work

Supplementary Figures

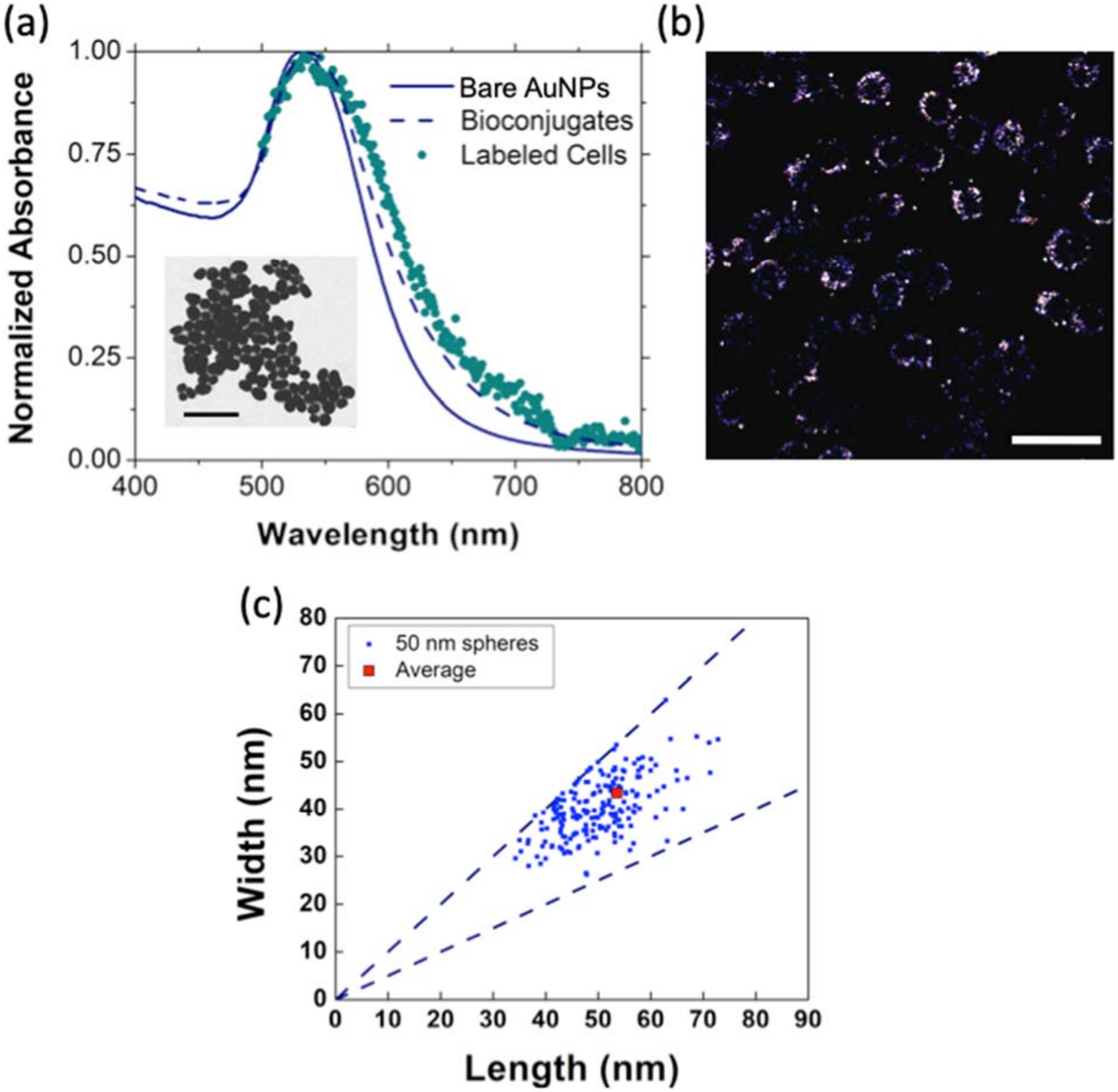

Supplementary Figure 1: Optical properties of gold nanospheroids labeled MDA-MB-468 epithelial breast cancer cells. (a) Normalized absorption spectra for both bare (blue line) and functionalized (bio-conjugated) nanoparticles (dotted blue line), and 3.6×10^6 labeled cells in suspension (green dots). Inset shows a representative SEM image of bare nanoparticles with a scale bar of 200 nm. (b) Cross-sectional multiphoton luminescence image of labeled cells in suspension obtained using 760 nm wavelength and 0.5 mW average powers. Cells were labeled at a concentration of 2×10^4 particles per cell. Scale bar corresponds to 25 µm. (c) Plot of lengths and widths of 200 gold nanoparticles as measured from the TEM images (**Supplementary Table 3**). Dashed lines represent aspect ratios (AR) of 1 and 2.

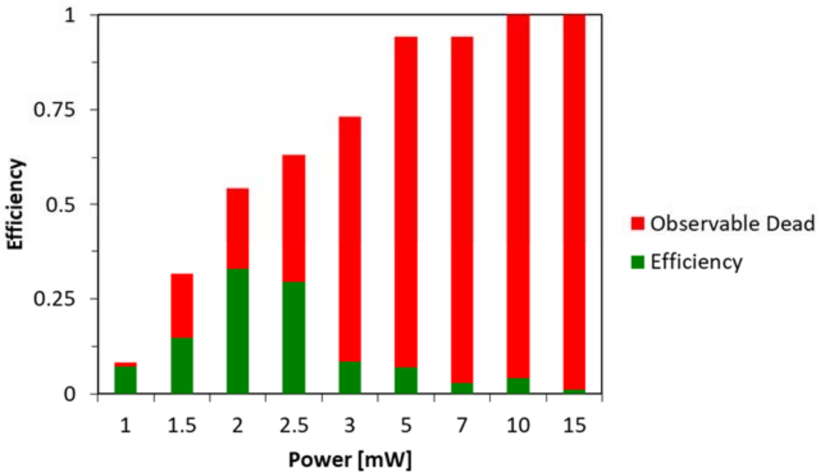

Supplementary Figure 2: Cell death after PLN with circularly polarized light. At higher powers (>2 mW), we find that the percentage of cells that remained viable (~2%) when exposed to circular-polarized laser pulses was significantly lower than the 5% that remained viable after linearly polarized irradiation.

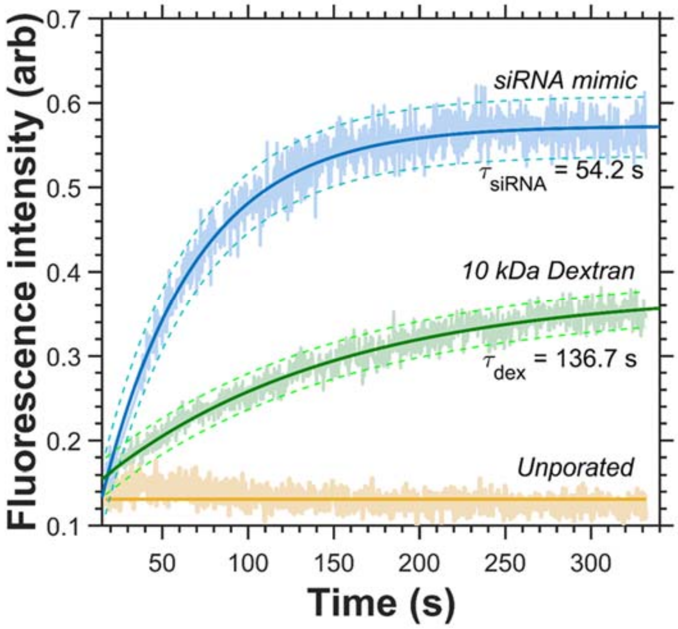

Supplementary Figure 3: Uptake kinetics of PLN-induced transient pore formation. The signal intensity of 10 kDa FITC-Dextran and siRNA mimics were monitored inside and outside single cells after irradiation with 30 scans of 2.6 mJ/cm^2 laser fluence. Exponential fits to the intensity rise were used to determine the hole-closure time, and are represented as solid lines. Dotted lines show prediction intervals at 95% confidence. Dextran diffuses slower than siRNA due to its larger size.

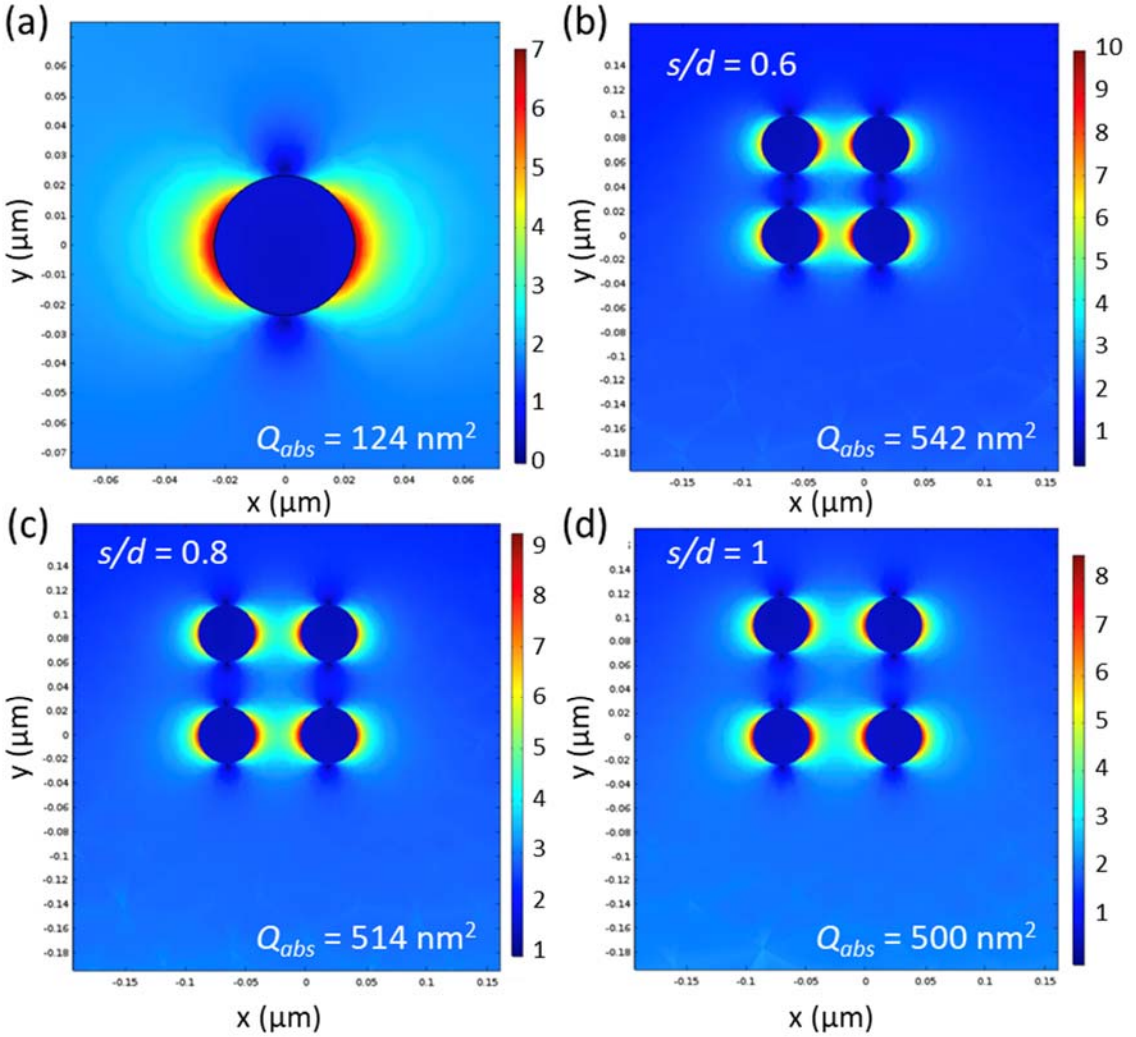

Supplementary Figure 4: **Simulation of the absorption cross-sections (*Q_{abs}*) and Poynting vector enhancements (*Q_{Poyn}*) in and around a 47 nm diameter single particle and a 4-particle cluster (*d* = 47 nm) in a water medium for various *s/d* values, excited with horizontally polarized 760 nm laser light.** (a) Poynting vector enhancement ($|S|/|S_0|$) around a single particle in water, showing over a 7-fold of maximum enhancement at the particle surface. (b,c,d) Poynting vector enhancement around a 4-particle cluster with packing factor of (b) *s/d* = 0.6 with a maximum enhancement of 10.0, (c) *s/d* = 0.8 with a maximum enhancement of 9, and (d) *s/d* = 1.0 with a maximum enhancement of 8.2, only varying by 10% between each case. These values result in an increase enhancement ranging from 1.43 (*s/d* = 0.6) to 1.2 (*s/d* = 1) over the 7-fold enhancement that a single particle experiences at the surface. A packing factor of 0.8 is taken for our simulations in the manuscript. For the Poynting vector simulations, we used a finite element model to solve Maxwell's equations in frequency domain. The complex permittivity of the gold nanoparticle is calculated using the Brendel-Bormann model[1]. Perfectly matched layer outside of the computational region is applied to absorb the scattered thermal radiation at the far-field in all directions. Also, non-uniform meshes are implemented to save computational time effectively and to produce convergence. The results showed a maximum enhancement of ~9, which is 1.29× over the 7 times enhancement that a single particle experiences at the surface.

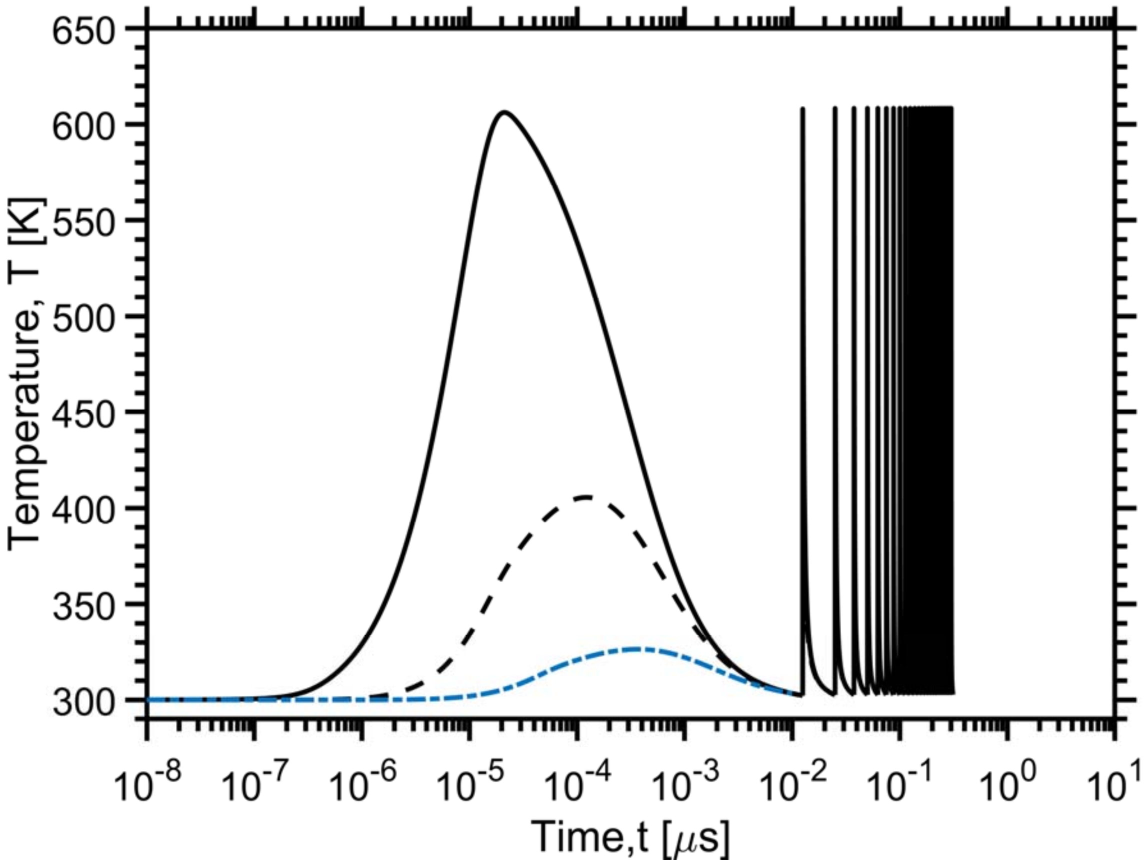

Supplementary Figure 5: Thermal simulations for our maximum operating average input fluence of 9.1 mJ/cm^2 (7 mw). The temperature dynamics of a cluster with 4 gold nanoparticles (d = 47 nm) and surrounding water at an average input fluence of 9.1 mJ/cm^2 (pulse energy of 87.5 pJ) over the total exposure time of a spot in the field of view during a single line scan, which comprises of 378 consecutive pulses. For conservative calculations, we assumed the particles are located at the laser's focal center and experience the highest local fluences, namely the peak fluences, which are twice the average input fluence. The black solid line represents gold lattice temperature. The black dashed line represents the temperature evolution of water near the particle interface where the water temperature reaches the highest values. The blue dashed line represents the temperature at the cluster-center and shows that there is no lingering thermal build-up between pulses at this highest fluence.

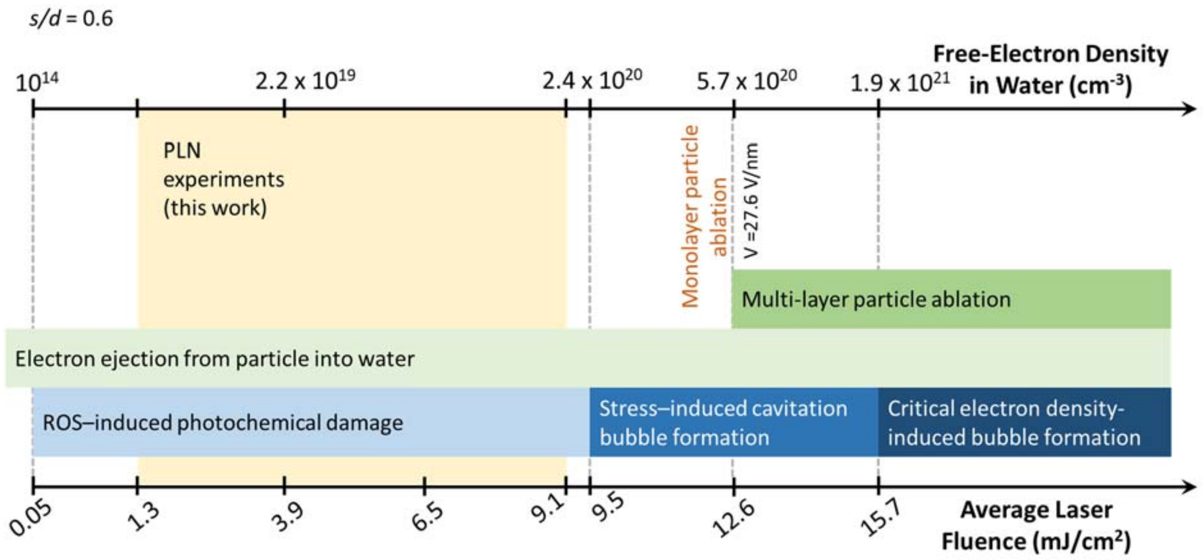

Supplementary Figure 6: Schematic describing the various regimes of PLN driven by a 760 nm, 270 fs laser pulse interacting with a 47 nm gold nanoparticle pair for a packing factor of *s/d* = 0.6. We ran the simulations for *s/d* = 0.6 as the lower bound from our literature analysis, with lower *s/d* values expected increasing the enhancement effects. Fluences used in the simulations assume the particles are located at the focal center, and therefore experience twice the average input fluence locally. The increased field enhancement effects obtained at an *s/d* of 0.6 did not change the overall photomodification processes involved in our model where we used *s/d* = 0.8 (Figure 6).

Supplementary Tables

Supplementary Table 1: Operating parameters for the literature surveyed in Figure 7 with femtosecond (fs) laser pulse widths. AuNS stands for gold nanospheres, ROS stands for reactive oxygen species.

Article	Pulse Fluence (mJ/cm^2)	Rep rate (kHz)	λ (nm)	Pulse duration		Nano-particles	Suggested mechanism of PLN
Davis 2013[2]	382	80,000	800	100	fs	None	ROS
Stevenson 2006[3]	80	80,000	800	120	fs	None	ROS
Suppato 2005[4]	70	80,000	830		fs	None	ROS, cavitation bubbles (high n)
Sacconi 2007[5]	50	95000	850	120	fs	None	ROS
Tirlapur 2001[6]	20	80,000	800	170	fs	None	ROS
Uchugonova 2008[7]	15	75,000	795	20	fs	None	Photoionization
Davis 2013[2]	4,584	1	800	100	fs	None	Photoionization cavitation bubbles
Bourgeois 2007[8]	500-4,100	1	780	220	fs	None	Photoionization cavitation bubbles
Watanabe 2004[9]	1200	1	800	150	fs	None	Plasma-mediated ablation
Shen 2005[10]	600	1	800	100	fs	None	Plasma-mediated ablation
Heisterkamp 2005[11]	600	1	790	230	fs	None	Plasma-mediated ablation
Schomaker 2015[12]	200	5	796	120	fs	200 nm AuNS	Off-resonant, plasma induced cavitation
Baumgart 2012[13]	80	1	800	45	fs	100 nm AuNS	Off-resonant, plasma induced cavitation
Minai 2013[14]	17-25	1	550	50	fs	20 nm AuNS	On-resonant, Photoionization bubbles+ ROS
Mazumder 2007[15]	14	80,000	800		fs	5 nm AuNS	Off-resonant, thermal cavitation suggested
Boutopoulos 2015[16]	50	0.003	800	45	fs	100 nm AuNS	Off-resonant, plasma induced cavitation
Bergeron 2015[17]	60-80	0.5	800	45	fs	100 nm AuNS	Off-resonant, plasma induced cavitation
**This work**	65	**80,000**	**760**	**270**	**fs**	**None**	**ROS**
**This work**	**1.3-9.1**	**80,000**	**760**	**270**	**fs**	**50 nm AuNS**	**Off-resonant, ROS-induced photo-chemical damage**

Supplementary Table 2: Operating parameters for the literature surveyed in Figure 7 with picosecond (ps) laser pulse widths. AuNS stands for gold nanospheres.

Article	Fluence (mJ/cm^2)	Rep rate (kHz)	λ (nm)	Pulse duration		Nano-particles	Suggested mechanism of PLN
Yao 2009[18]	100	10	527	35	ps	15, 30 nm AuNS	On-resonant, photothermal bubbles
Kalies 2013[19]	64	20.25	532	850	ps	80,150, 200 ,250 nm AuNS	On-resonant, mechanism not explored (possibly Photoionization/ thermal bubbles)
Heinemann 2014[20]	42	20.25	532	850	ps	200 nm AuNS	On-resonant, photothermal bubbles
Kalies 2014[21]	30	20.25	532	850	ps	200 nm AuNS	On-resonant, Photoionization or photothermal bubbles
Heinemann 2013[22]	20	20.25	532	850	ps	200 nm AuNS	On-resonant, photothermal bubbles or photoionization

Supplementary Table 3: **Summary of measured physical properties of nanospheres, plotted in Supplementary Figure 1c.**

Particle	Length x Width (nm)	Ellipticity	Size Distribution	Zeta Potential (mV)	Q_{ext}
**Gold nano-spheres**	54 ± 6 × 44 ± 4<br>Eq. vol. sphere having *d* = 47 ± 5 nm	1.24 ± 0.13	10 %	-27.6 ± 2.2	3.56

Supplementary Notes

Supplementary Note 1: Particle characterization

Here we provide a full characterization of our gold nanoparticles. We measured the linear absorption of each particle set using UV-Vis Spectroscopy (Beckman-Coulter) and cuvettes having path length 0.5 cm. The normalized linear absorbance spectrum is presented in **Supplementary Figure 1a**. The particles exhibit a narrow absorption peak centered at 531 nm with 70 nm FWHM, which is characteristic of dipolar resonance. Broadening of the plasmon band results from the larger size distribution and ellipticity.

A gold nanoparticle concentration of 3.4 x 10^{10} particles/mL was estimated from the UV-Vis spectra with less than a 6% error using

$$N_{np} = \frac{2.303A}{\pi r_s r_i Q_{ext} d_0} \quad , \tag{1}$$

where Q_{ext} is the theoretically modeled extinction efficiency, A is the peak absorbance as found from the UV-Vis spectrum, r_s and r_i are the STEM measured radii in the short and long axis of the spheroid, respectively, and d_0 is the cuvette path length. Due to the spheroidal shape of the generated particles, Discrete Dipole Approximation (DDA) performed by Dr. R.K. Harrison was used to model the extinction efficiency[23–27]. DDA is a well-established technique for simulating the optical properties of non-spherical colloidal nanoparticles, because it can be utilized to account for particle size distribution and orientation. Non-spherical particle geometries can be simulated because the overall structure is organized via the superposition of small cube sections; across each cube, the induced dipole polarization from the incident light is assumed to be constant.

DDA model parameters to determine Q_{ext}: The spheroidal shape was organized such that there were 128 dipoles across the short axis, which is equivalent to each dipole representing a 0.04 nm^3 volume. The complex gold refractive index at the irradiation wavelength was obtained from experimentally determined refractive index values for bulk gold and then corrected for size-related surface damping according to the Drude equation with a modified damping constant. Optical properties were simulated for isolated particles in a water environment at intervals of 20 nm across a 400 to 900 nm wavelength range.

Accounting for orientation effects with respect to polarization[a] and size distribution[b] of the sample, an average extinction coefficient of Q_{ext} = 3.56 was found. If the spheroidal shape were neglected, the concentration would have been over-estimated.

---

[a] Spheroids were simulated at four orientations with respect to the incident light polarization and propagation directions. The long axis of the spheroid is simulated along the x, y, z and [1 1 1] directions with light propagation along the z direction and polarization along the x axis.

[b] Due to the broad distribution of the GNS530 sample, simulations were performed at plus/minus on standard deviation in the spheroid long axis and constant width.

The physical characteristics of our nanoparticles are summarized in **Supplementary Table 3**. The physical dimensions of the generated nanoparticles were characterized with high-resolution field emission scanning transmission electron microscopy (STEM; Hitachi S-5500); R. K. Harrison obtained particle images. A 2µL aliquot from the stock solution was dried onto a copper grid (Cu-400CN; GridTech) at 120 °C, washed via a series of water droplets, and allowed to air dry. The inset of **Supplementary Figure 1a** provides a representative image of the generated particles at 150,000 x magnification. Image analysis of the particles were performed using ImageJ. We found that our batch of nanoparticles measured 54 ± 6 nm × 44 ± 4 nm (length × width, equivalent volume sphere *d* = 47 ± 5 nm). **Supplementary Figure 1c** provides a size distribution scatter plot for a sample set of 200 particles. The nanoparticles have an aspect ratio between 1 and 2 with an ellipticity of 1.24 ± 0.13, which is consistent with the formation of spheroidal particles, and a size distribution of approximately 10%.

The surface charge of our gold sol was determined through zeta potential measurements with a ZetaPlus® (Brookhaven Instruments Corporation). The nanoparticles were re-suspended at a concentration of 28 pM in a 1 mM KCl solution (pH 7.5). Ten zeta potential readings were obtained and averaged, revealing the GNS530 gold sol is stable, having an average surface charge of -27.6 ± 2.2 mV. Zeta potential indicates the degree of repulsion between adjacent particles, with potentials ~ -11 to -20 mV being the threshold below which agglomeration occurs[28,29]. Particle stability, i.e. monodispersity, is supported by both the high zeta potential and relatively narrow spectral bandwidth.

Supplementary Note 2: Characterizing Transient Pore Formation

To quantify PLN-induced pore formation, we monitored the uptake of 10-kDa Dextran and a siRNA mimic (14.9 kDa) after PLN using two-photon microscopy in separate experiments. A pre-determined concentration of fluorescent probe (25 mM Dextran; 20 pM siRNA mimic) was added to the extracellular solution prior to irradiation. The cells were irradiated with 30 scans of 2.6 mJ/cm^2 incident fluence (2 mW power), chosen for its high rate of fluorescent probe retention. **Supplementary Figure 3** presents the time dependent variation of extracellular and intracellular fluorescence signal levels as measured from the two-photon images of irradiated cells. Within the whole FOV, close to 75% of cells experienced material influx. Cells that were permeabilized showed an exponential increase in fluorescence intensity immediately after targeting. The fluorescence intensity then plateaued, indicating pore closure, or continued to increase towards the intensity values of the surrounding media, indicating permanent membrane dysfunction.

A simple diffusion-based model was used to estimate the PLN-produced hole radius[2]. Briefly, we assume that hole with a constant radius *r* is produced in the cell membrane, and is of course, much smaller than the cell radius. The increase in dye concentration within the cell, φ can then be represented as an exponential rise using Fick's law of diffusion,

$$\varphi(t) = \varphi_0 \left(1 - \exp\left({-t}/{\tau_{pore}} \right)\right) , \tag{2}$$

where φ_0 is the final dye concentration within the cell and τ_{pore} is the diffusive time constant for the hole. Knowing the diffusion constant *D* for the molecule, and the membrane thickness *e*, the effective radius of the hole, *r*, can be determined from the expression for τ ,

$$\tau_{pore} = \frac{eV_{cell}}{D(\pi \cdot r^2)} , \tag{3}$$

where V_{cell} is the volume of the cell. We fit the measured rise of fluorescence within the optoporated cells to **Eq. 2** to determine τ_{pore} , and estimate the hole radius *r* from **Eq. 3**. We found that the fluorescence intensity within optoporated cells increased with a rise time (τ_{pore}) of 136.7 ± 5.8 s for Dextran and 54.2 ± 2.0 s for siRNA, with comparable hole radii of 17.8 ± 4.9 nm and 17.9 ± 4.8 nm, respectively. The similar hole-radii estimates obtained using different transfection agents (with different *D*) points to the validity of the 1D diffusion model. The pore radius should allow us to transfect Dextran up to 2,000 kDa at 2.6 mJ/cm^2, with a theoretical diameter of 12 nm[30], albeit at slower diffusion rates.

In a different experiment, to verify if the intensity plateau corresponded with pore closure, *i.e.*, membrane healing, the FITC-Dextran probe was added to the extracellular solution 180 ($\tau_{pore,0.73}$) and 240 ($\tau_{pore,0.83}$) seconds after PLN. While a small percentage of cells still incurred dye influx at the 180 s mark, no influx was found beyond 240 s post irradiation. Comparable closure times and pore radii were observed in optoporation experiments using tightly focused NIR, femtosecond laser pulses at high repetition rates in cells sans nanoparticles[2,31].

Supplementary Note 3: First order model for photoemission from particles and free electron generation in water

To explain our experimental observations and provide a comprehensive and quantitative description of electron emission and nanoparticle ablation using ultrashort laser pulses, we apply the generalized Fowler-DuBridge theory[32] to plasmonic nanospheres. We find particle Poynting vector enhancements using Finite Difference Frequency Domain solution of Maxwell's equations for a 4-particle cluster of gold spheres with *d* = 47 nm and *s/d* =0.8 (**Supplementary Figure 4**) while electron temperature evolutions are determined using the two-temperature model for laser-metal heating.

Conduction band electrons may be ejected to the surrounding medium from a material at elevated temperatures when their kinetic energies exceed the work function. Electron emission due to thermal kinetic energy, referred to as thermionic emission, exists at all temperatures down to zero Kelvin. In addition to thermionic emission, electrons may be released from surfaces by exposure to high intensity fields at the surface[33,34]. High intensity field strengths at a material surface may result in sufficient photon density so that one or more photons are absorbed by conduction band electrons at the material surface, causing their surface-normal kinetic energy components to exceed the barrier energy necessary for electron escape. For nanoparticles interacting with ultrafast laser pulses, the high absorption cross sections of plasmonic particles coupled with short pulses create conditions favorable for both thermionic and multi-photon emission processes.

The generalized Fowler-DuBridge theory of photoemission current density (J), as described by Bechtel *et al.*[34] incorporates all orders of emission processes as a series expansion with activation energy being a function of electron temperature (T_e), material work function (ϕ), and multiphoton process order (n):

$$\boldsymbol{J_n} = a_n \left(e/_{h\nu} \right)^n A_0 I(\boldsymbol{r},t)^n (1-R)^n T_e(\boldsymbol{r},t)^2 F\left(\frac{nh\nu - \phi}{k_B T_e(\boldsymbol{r},t)} \right), \tag{4}$$

where A_0 is the Richardson coefficient (120 A/cm^{2}K^2), a_n is an order-specific constant, and F is the Fowler function. Thermionic emission is assumed to be a zeroth order process, and described by

$$J_0 = A_0 T_e^2 \exp\left(-\frac{\phi}{k_B T_e} \right) . \tag{5}$$

A slightly modified version of this theory describes specific $\boldsymbol{J_n}$[35,36] for photoemission current for three-photon process (n=3)

$$J_3 = 2c\left(\frac{k_B T_e(t)}{3h\nu - \phi} \right)^2 I(r,t)^3 F\left(\frac{3h\nu - \phi}{k_B T_e(t)} \right), \tag{6}$$

with

$$c \propto \frac{a_3}{2}\left(\frac{e}{\hbar\omega} \right)^3 \left(\frac{3\hbar\omega - \phi}{k_B} \right)^2 . \tag{7}$$

Here c describes the three-photon ionization cross-section and is set at 10^{-7} Acm^4/MW^3 and ϕ is

set to 3.72 eV[35,36]. The surface reflectivity (*R*) is not considered since the source term here (I) accounts for the local intensity in the near-field through Q_{Poyn}. We can then calculate the total number of emitted electrons (*N*) by integrating the current density $J_0 + J_3$ over the particle surface at each time step. To pursue this calculation, we first need to define the local intensity, which is modified by the particle near-field enhancement according to

$$I(t) = Q_{Poyn} \frac{2F}{\tau_p} \sqrt{\frac{\ln 2}{\pi}} \exp\left[-4 \ln 2 \cdot \left(\frac{t}{\tau_p} \right)^2 \right]. \tag{8}$$

Gauss' Law along with the assumption of uniform charge distribution within the particle yields the induced electric field strength as a function of radial position, *r*, within the particle,

$$E(r) = \frac{rNe}{3\varepsilon V_{np}} \tag{9}$$

giving

$$E = \frac{d}{6\varepsilon V_{np}} \int_t \int_A (\boldsymbol{J_0} + \boldsymbol{J_3}) dt dA \tag{10}$$

where *d* is the particle diameter, ε is the nanoparticle permittivity, and V_{np} is the volume of the nanoparticle. When the induced field at the particle surface reaches a critical value (E_{cr}) where the electrostatic repulsion of the lattice exceeds the lattice binding strength, the particle begins to ablate. Bulgakova and co-workers[36] estimated this value for gold to be 2.76 × 10^{10} V/m. The validity of a uniform charge distribution is questionable for pulse durations on the order of electron-electron relaxation timescale of femtoseconds. However, this assumption will produce a conservative estimate of ablation initiation. Fowler-Nordheim type emission may be ruled out by examining the Anisimov parameter for laser intensities and wavelengths used here[37].

In the case of multi-particle interactions, the Q_{Poyn} associated with the particle will vary with the extent of inter-particle coupling. Our simulations showed a 1.29 × increase in the Poynting vector enhancement for the 4-particle cluster over a single particle (**Supplementary Figure 4**), with a maximum single particle enhancement of 7 at the particle surface. This increased enhancement per particle was used when simulating the photoemission rates from a single particle. Likewise, the increased near-fields and absorption was incorporated in our free electron generation model for water, discussed below.

The free-electron generation/plasma formation in water is handled similar to Vogel *et al.*[38], where water is treated as a dielectric medium with an ionization potential $\tilde{\Delta}$ of 6.5 eV. We expect most of the free electron generation to take place within this shell for a given particle and ignore free electron diffusion into the volume from surrounding media. We do not expect the plasma from neighboring particles to interact over the duration of the pulse since plasma diffusion is quite slow. As such, simulations are performed for a single particle, with field enhancements accounting for multi-particle interactions. The time evolution of electron density ρ due to laser irradiance is calculated using a modified version of the rate equation of the generic form[38–41]

$$\frac{d\rho}{dt} = -\nabla \bullet J + \eta_{photo} + \eta_{casc}\rho - \eta_{diff}\rho - \eta_{rec}\rho^2 . \quad (11)$$

The first term represents the free electrons generated from the particle and represents the divergence of the photocurrent from **Eqs. 5 and 6**. The particle photoemission rate is calculated using the Divergence theorem,

$$\nabla \bullet J = \frac{\oint_A J dA}{dV} . \quad (12)$$

Here we assume dV to be a volume shell extending out from the surface of the particle. The thickness of the shell is determined by the expected diffusion length of ejected electrons and is given by[42]

$$l_D = \left(Dt_i\right)^{1/2} \quad (13)$$

for time t_i. Here D is the diffusion coefficient of the electrons and is given by $D = \tau k_B T_e / (m_0)$, with τ the mean free time between collisions (5 fs for plasma in gaseous plasma in water[43]). Diffusion is assumed to occur radially in one dimension. The diffusion length is used to recalculate dV at each time step, and the concentration of photoemitted electrons is calculated using **Eq. 12** assuming the concentration to be constant over l_D.

The η_{photo} term refers to the free electrons generated by the ambient laser field in the vicinity of the nanoparticle due to multiphoton ionization or tunneling. The time averaged ionization rate for a field with angular frequency ω and intensity I was derived by Keldysh for condensed media[44]. For the limiting case of multi-photon ionization where tunneling is ignored, it has the form[42]

$$\eta_{photo} = \left(\frac{d\rho}{dt}\right)_{photo} = \frac{2\omega}{9\pi}\left(\frac{m\omega}{\hbar}\right)^{3/2}\left(\frac{e^2}{16m\tilde{\Delta}\omega^2 c\varepsilon_0 n} I\right)^k \exp\left(2k\right)\Phi\left(\sqrt{2k - \frac{2\tilde{\Delta}}{\hbar\omega}}\right) \quad (14)$$

where $\Phi(x)$ represents the Dawson probability integral,

$$\Phi(x) = \exp(-x^2)\int_0^\infty \exp(y^2)dy . \quad (15)$$

Here, ω is the circular frequency of incoming light, $\hbar$ is the reduced Plank constant, e represents the electron charge, c is the vacuum speed of light, ε_0 is the permittivity of free space, and n_0 is the refractive index of the medium (water) at frequency ω. The number of photons needed to overcome the bandgap is given by $k = \left\langle \tilde{\Delta}/\hbar\omega + 1 \right\rangle$ and m represents the exciton reduced mass, defined as $1/m = 1/m_c + 1/m_v$ where m_c and m_v are the masses of the conduction band electron and hole in the valance band. The intensity of incoming laser irradiation is defined as I, with a circular frequency ω.

The cascade contribution must take into account the need for seed electrons to initiate the cascade process through inverse Bremsstrahlung absorption and impact ionization. Likewise, the

process rate must account for ionization time $\tau_{ion} = n.\tau$, determined by the number of photons (*n*) that need to be absorbed to acquire sufficient energy for impact ionization, and the mean free time τ (5 fs for plasma in gaseous plasma in water[43]).

Therefore, the cascade ionization rate is determined using the electron density at time $t_{ret} = t - \tau_{ion}$ assuming a 50% probability of having a start electron in the focal volume, with the first order approximation expressed as

$$\left(\frac{d\rho}{dt}\right)_{casc} = \begin{cases} \dfrac{\eta}{1+\eta t_{ret}} & for\ \rho_c V \geq 0.5 \\ 0 & for\ \rho_c V < 0.5 \end{cases}, \tag{16}$$

where η represents the ionization rate per electron participating in the cascade[42],

$$\eta = \frac{1}{\omega^2\tau^2+1}\left(\frac{e^2\tau}{cn_0\varepsilon_0 m_c(3/2)\tilde{\Delta}}I - \frac{m_c\omega^2\tau}{M}\right), \tag{17}$$

where *M* is the mass of the water molecule.

Free electrons are lost from the interaction volume via diffusion out of the volume *V* and through recombination. In our case, we look at a small volume at the enhancement 'pole' beside the particle, where the enhancement in the particle is at its maximum. Assuming a spherical voxel of radius 125 nm, the resulting ionization rate due to diffusion is given by[38]

$$\left(\frac{d\rho}{dt}\right)_{diff} = \frac{\tau E_{av}}{3m\Lambda^2}\cdot\rho_c\ , \tag{18}$$

where the characteristic diffusion length is Λ. Here we assume 1D diffusion radially across the voxel in one direction and use the diffusion length for a spherical shell given by $(1/\Lambda^2) = \left\{\frac{\pi}{R_1\left(R_2/R_1\right)-1}\right\}^2$, where R_1 and R_2 are the inner and outer radii.

The average kinetic energy of the free electrons is calculated as the average of the energy of photo-emitted electrons from the particle[41] ($1.5k_BT_e$), photoemitted electrons from water ($0.5\tilde{\Delta}$) and the energy of the electron produced by the collision ionization process ($2.5\tilde{\Delta}$). We assume that no diffusion from the neighboring voxel in water. We can expect the photoemitted electrons from the particle to still predominantly remain within the 5 nm voxel width over the duration of the pulse, considering that our electron temperatures are less than 0.5 eV. Simulations and experiments have shown plasma fronts moving under 20 nm for high density plasmas with electron temperatures in the 10-100 eV range[45,46]. For the recombination rate, we used an empirical value obtained by Docchio *et al.*[47]

$$\left(\frac{d\rho}{dt}\right)_{rec} = 2\times10^{-9}\ cm^3/s\times\rho_c^2\ . \tag{19}$$

A fourth order Runga-Kutta scheme was used to numerically solve the rate equation for various input laser intensities. The various effects of particle photoemission and free electron generation in water are represented schematically in **Figure 6** in the main text.

While our analysis builds on a widely used model for free electron generation in water, recent work has further improved on the model through a detailed rework based on a more accurate estimate of the band gap of water, which was updated to 9.5 eV[48]. The rate equation for strong field ionization was also updated to account for excitation to intermediate solvated states in the conduction band, and direct photoionization. The value of τ was also revised down to 0.9 fs (1,050 nm). In our case, our experimental threshold is portrayed with a bandgap that is too low by using a larger collision time is increased. Our model, in essence, provides a sense of the range of electron densities over which PLN operates for our input fluences.

Supplementary References

1. Rakić, A. D., Djurišić, A. B., Elazar, J. M. & Majewski, M. L. Optical properties of metallic films for vertical-cavity optoelectronic devices. *Appl. Opt.* **37**, 5271 (1998).

2. Davis, A. a, Farrar, M. J., Nishimura, N., Jin, M. M. & Schaffer, C. B. Optoporation and genetic manipulation of cells using femtosecond laser pulses. *Biophys. J.* **105**, 862–71 (2013).

3. Stevenson, D. *et al.* Femtosecond optical transfection of cells: viability and efficiency. *Opt. Express* **14**, 7125–7133 (2006).

4. Supatto, W. *et al.* In vivo modulation of morphogenetic movements in Drosophila embryos with femtosecond laser pulses. *Proc. Natl. Acad. Sci. U. S. A.* **102**, 1047–52 (2005).

5. Sacconi, L. *et al.* In vivo multiphoton nanosurgery on cortical neurons. *J. Biomed. Opt.* **12**, 050502 (2007).

6. Tirlapur, U. K., König, K., Peuckert, C., Krieg, R. & Halbhuber, K. J. Femtosecond near-infrared laser pulses elicit generation of reactive oxygen species in mammalian cells leading to apoptosis-like death. *Exp. Cell Res.* **263**, 88–97 (2001).

7. Uchugonova, A., König, K., Bueckle, R., Isemann, A. & Tempea, G. Targeted transfection of stem cells with sub-20 femtosecond laser pulses. *Opt. Express* **16**, 9357–9364 (2008).

8. Bourgeois, F. & Ben-Yakar, A. Femtosecond laser nanoaxotomy properties and their effect on axonal recovery in C. elegans. *Opt. Express* **15**, 8521 (2007).

9. Watanabe, W. *et al.* Femtosecond laser disruption of subcellular organelles in a living cell. *Opt. Express* **12**, 4203–13 (2004).

10. Shen, N. *et al.* Ablation of cytoskeletal filaments and mitochondria in live cells using a femtosecond laser nanoscissor. *Mech. Chem. Biosyst.* **2**, 17–25 (2005).

11. Heisterkamp, A. *et al.* Pulse energy dependence of subcellular dissection by femtosecond laser pulses. *Opt. Express* **13**, 3690–6 (2005).

12. Schomaker, M. *et al.* Characterization of nanoparticle mediated laser transfection by femtosecond laser pulses for applications in molecular medicine. *J. Nanobiotechnology* **13**, 10 (2015).

13. Baumgart, J. *et al.* Off-resonance plasmonic enhanced femtosecond laser optoporation and transfection of cancer cells. *Biomaterials* **33**, 2345–50 (2012).

14. Minai, L., Yeheskely-Hayon, D. & Yelin, D. High levels of reactive oxygen species in gold nanoparticle-targeted cancer cells following femtosecond pulse irradiation. *Sci. Rep.* **3**, 1–7 (2013).

15. Mazumder, A. & Shivashankar, G. V. Gold-nanoparticle-assisted laser perturbation of chromatin assembly reveals unusual aspects of nuclear architecture within living cells. *Biophys. J.* **93**, 2209–2216 (2007).

16. Boutopoulos, C. *et al.* Cell-specific optoporation with near-infrared ultrafast laser and functionalized gold nanoparticles. *J. Biophotonics* **7**, 17836–17847 (2015).

17. Bergeron, E. *et al.* Cell-specific optoporation with near-infrared ultrafast laser and functionalized gold nanoparticles. *Nanoscale* **7**, 17836–17847 (2015).

18. Yao, C., Qu, X., Zhang, Z., Hüttmann, G. & Rahmanzadeh, R. Influence of laser parameters on nanoparticle-induced membrane permeabilization. *J. Biomed. Opt.* **14**, 054034 (2009).

19. Kalies, S. *et al.* Plasmonic laser treatment for Morpholino oligomer delivery in antisense applications. *J. Biophotonics* **9**, 1–9 (2013).

20. Heinemann, D. *et al.* Delivery of proteins to mammalian cells via gold nanoparticle mediated laser transfection. *Nanotechnology* **25**, 245101 (2014).

21. Kalies, S. *et al.* Enhancement of extracellular molecule uptake in plasmonic laser perforation. *J. Biophotonics* **7**, 474–482 (2014).

22. Heinemann, D. *et al.* Gold Nanoparticle Mediated Laser Transfection for Efficient siRNA Mediated Gene Knock Down. *PLoS One* **8**, 1–9 (2013).

23. Draine, B. T. & Flatau, P. J. Discrete-Dipole Approximation For Scattering Calculations. *J. Opt. Soc. Am. A* **11**, 1491 (1994).

24. Johnson, P. B. & Christy, R. W. Optical Constants of the Noble Metals. *Phys. Rev. B* **6**, 4370–4379 (1972).

25. Palik, E. D. *Handbook of optical constants of solids*. (Academic Press, 1998).

26. Scaffardi, L. B. & Tocho, J. O. Size dependence of refractive index of gold nanoparticles. *Nanotechnology* **17**, 1309–1315 (2006).

27. Coronado, E. A. & Schatz, G. C. Surface plasmon broadening for arbitrary shape nanoparticles: A geometrical probability approach. *J. Chem. Phys.* **119**, 3926–3934 (2003).

28. Lu, G. W. & Gao, P. Emulsions and Microemulsions for Topical and Transdermal Drug Delivery. in *Handbook of Non-Invasive Drug Delivery Systems* 59–94 (Elsevier, 2010). doi:10.1016/B978-0-8155-2025-2.10003-4

29. Bhattacharjee, S. DLS and zeta potential – What they are and what they are not? *J. Control. Release* **235**, 337–351 (2016).

30. Haller, W. Critical permeation size of dextran molecules. *Macromolecules* **10**, (1977).

31. Peng, C., Palazzo, R. E. & Wilke, I. Laser intensity dependence of femtosecond near-infrared optoinjection. *Phys. Rev. E - Stat. Nonlinear, Soft Matter Phys.* **75**, (2007).

32. DuBridge, L. A. Theory of the Energy Distribution of Photoelectrons. *Phys. Rev.* **43**, 727–741 (1933).

33. Logothetis, E. M. & Hartman, P. L. Laser-Induced Electron Emission from Solids: Many-Photon Photoelectric Effects and Thermionic Emission. *Phys. Rev.* **187**, 460–474 (1969).

34. Bechtel, J. H., Lee Smith, W. & Bloembergen, N. Two-photon photoemission from metals induced by picosecond laser pulses. *Phys. Rev. B* **15**, 4557–4563 (1977).

35. Bulgakova, N. M., Stoian, R., Rosenfeld, A., Hertel, I. V. & Campbell, E. E. B. Electronic transport and consequences for material removal in ultrafast pulsed laser ablation of materials. *Phys. Rev. B* **69**, 054102 (2004).

36. Bulgakova, N. M. *et al.* A general continuum approach to describe fast electronic transport in pulsed laser irradiated materials: The problem of Coulomb explosion. *Appl. Phys. A Mater. Sci. Process.* **81**, 345–356 (2005).

37. Girardeau-Montaut, J. P. & Girardeau-Montaut, C. Theory of ultrashort nonlinear multiphoton photoelectric emission from metals. *Phys. Rev. B* **51**, 13560–13567 (1995).

38. Vogel, A., Noack, J., Hüttman, G. & Paltauf, G. Mechanisms of femtosecond laser nanosurgery of cells and tissues. *Appl. Phys. B* **81**, 1015–1047 (2005).

39. Haering, S. Nanoparticle Mediated Photodistruption. (University of Texas at Austin, 2010).

40. Boulais, E., Lachaine, R. & Meunier, M. Plasma mediated off-resonance plasmonic enhanced ultrafast laser-induced nanocavitation. *Nano Lett.* **12**, 4763–9 (2012).

41. Zhao, X. & Shin, Y. C. A two-dimensional comprehensive hydrodynamic model for femtosecond. (2012). doi:10.1088/0022-3727/45/10/105201

42. Kennedy, P. K. A first-order model for computation of laser-induced breakdown thresholds in ocular and aqueous media. I. Theory. *IEEE J. Quantum Electron.* **31**, 2241–2249 (1995).

43. Bataller, A., Kappus, B., Camara, C. & Putterman, S. Collision time measurements in a sonoluminescing microplasma with a large plasma parameter. *Phys. Rev. Lett.* **113**, 1–5 (2014).

44. Keyldesh, L. V. Ionization in the field of a strong Electromagnetic wave. *Sov. Phys. JETP* **47**, 1307–1314 (1964).

45. Zhao, X. & Shin, Y. C. Coulomb explosion and early plasma generation during femtosecond laser ablation of silicon at high laser fluence. *J. Phys. D. Appl. Phys.* **46**, (2013).

46. Andreev, N. E., Veisman, M. E., Efremov, V. P. & Fortov, V. E. The Generation of a Dense Hot Plasma by Intense Subpicosecond Laser Pulses. *High Temp.* **41**, 594–608 (2003).

47. Docchio, F., Regondi, P., Capon, M. R. & Mellerio, J. Study of the temporal and spatial dynamics of plasmas induced in liquids by nanosecond Nd:YAG laser pulses. 1: Analysis of the plasma starting times. *Appl. Opt.* **27**, 3661–8 (1988).

48. Linz, N., Freidank, S., Liang, X.-X. & Vogel, A. Wavelength dependence of femtosecond laser-induced breakdown in water and implications for laser surgery. *Phys. Rev. B* **94**, 024113 (2016).
